# A Cross-Country Network Analysis of Resilience Systems in Young Adults

**DOI:** 10.1177/21676968221090039

**Published:** 2022-05-02

**Authors:** Philip Jefferies, Jan Höltge, Jessica Fritz, Michael Ungar

**Affiliations:** 1 Faculty of Health, Resilience Research Centre, 3688 Dalhousie University, Halifax, NS, Canada; 2 Department of Psychiatry, Addenbrooke’s Hospital, 2152 University of Cambridge, Cambridge, UK

**Keywords:** resilience, adaptation, cross-cultural, network modelling, graph theory, multisystemic, cross-country

## Abstract

Multisystemic resilience has been conceptualised as involving a constellation of protective factors which operate at different levels to promote adaptation and thriving despite experiences of adversity. We used network modelling to discover how protective factors at two different systemic levels (intrapersonal strengths and social-ecological resources) interrelate, drawing on survey data from 5283 emerging adults (*M* = 24.53 years; 52% female) in Brazil, China, Indonesia, Russia, Thailand, the US and Vietnam. Results indicated that the level of connectivity within and between protective factor levels was similar between the countries, but that there was substantial variation in the specific interrelations among protective factors (both within and between levels), including the presence of some country-specific negative interrelations between protective factors at different levels. The findings support the importance of cultural context in studies of resilience, with implications for the development of appropriate resilience-building interventions for this age group.

Emerging adulthood (i.e. age 19–29) is a distinct and important developmental period associated with significant transitions across various life domains, including in living arrangements, employment and relationships (Arnett, 2000; Arnett et al., 2010). These transitions are also associated with heightened instability and uncertainty, which can provoke stress (Arnett, 2007; Lane et al., 2017), and have been linked to the greater prevalence of mental health disorders than are found in any other age group (Kessler & Walters, 1998; LeBlanc et al., 2020). Although much attention has been given to risk factors associated with this period (see Newcomb-Anjo et al., 2017; Schwartz & Petrova, 2019), the proportion of emerging adults who appear to demonstrate good wellbeing and an absence of psychopathology despite the experience of stress and trauma has led to an interest in their resilience (Burt & Paysnick, 2012; Madewell & Ponce-Garcia, 2016; Theron et al., 2020). In this study, we sought to explore the resilience systems of emerging adults around the world.

Resilience has been conceptualised in many ways over the years (see Chmitorz et al., 2018; Windle, 2011), but may be broadly defined as the capacity for maintaining or recovering functioning in the context of exposure to significant adversity (Masten et al., 2003; Ungar, 2019). Trait approaches were among the early conceptualisations which likened resilience to a singular personality quality like ‘hardiness’, which was thought to explain why some individuals would thrive in contexts where suboptimal outcomes were expected (Block & Block, 1980). As an outcome, resilience has been defined as a pattern of stable and healthy functioning following adversity (Bonanno et al., 2011), where states considered indicative of ‘being resilient’ (van Breda, 2018) may be reflected by internal accounts (e.g. good wellbeing, low stress and life satisfaction) or external measures (e.g. academic success and workplace engagement). More recently, resilience has been conceptualised as a dynamic process of positive adaptation following the experience of significant adversity (Bonanno et al., 2015; Bonanno & Diminich, 2013; Egeland et al., 1993; Windle, 2011). These processual accounts draw attention to the interaction of multiple modifiable factors within individuals and those existing in their environment which contribute to variable trajectories as individuals negotiate, adapt to, or otherwise manage sources of stress and trauma (Afifi & MacMillan, 2011; Benzies & Mychasiuk, 2009; Fritz, De Graaff, et al., 2018; Kumpfer, 2002; Masten, 2001).

Multisystemic modelling of resilience has brought these protective factors into relief, indicating that they can be found at different levels, ranging from the biological (e.g. the health of one’s microbiome; Relman, 2012; Rook et al., 2013) and psychological (e.g. patterns of attribution that avoid self-blame; Rubenstein et al., 2016) to social (e.g. supportive peer networks), cultural (e.g. participation in traditional practices) and environmental (Brown & Westaway, 2011; Ungar & Theron, 2020). However, many protective factors continue to be studied in isolation, despite increasing recognition of their systemic interactions (Fergus & Zimmerman, 2005; Fritz, De Graaff, et al., 2018; Masten, 2011).

Network studies present an insightful means to gain a greater understanding of the relative interactivity of protective factors implicated in processes of resilience at multiple systemic levels. There has been a proliferation of network studies in the social sciences recently, many of which can be found in the field of psychopathology (Contreras et al., 2019). In these studies, models of psychological disorders have presented patterns of connected symptom structures that are associated with consistent behavioural profiles or psychological syndromes (Borsboom, 2017; Borsboom & Cramer, 2013). The underlying feature of network studies when used in this way is that symptoms are not thought to reflect underlying mental disorders but in fact constitute them (McNally, 2016). In a similar way, resilience networks can help to reveal the complex interplay between protective factors and help to identify potential patterns of activation, deactivation, independence and dependence among factors (Hevey, 2018; van Bork et al., 2018).

Some network studies of resilience are beginning to produce compelling findings along these lines (Briganti & Linkowski, 2019; de Hallen et al., 2020; Fritz, Fried, et al., 2018; Hoorelbeke et al., 2016; Lunansky et al., 2021; Thoma et al., 2020), but tend to be limited to exploring protective factors at just one level (e.g. psychological factors). In contrast, a network study of multisystemic resilience could also indicate the extent to which protective factors at one systemic level (e.g. peer support) are associated with those of another co-occurring level (e.g. cognitions that support a positive future orientation), and those which remain independent. One study that has already explored such ‘bridges’ (Jones et al., 2019) between protective factors was conducted by Höltge et al. (2021), who examined samples of adolescents and found that interconnectivity between individual (e.g. problem solving) and caregiver-related factors (e.g. feeling safe with caregivers) was weaker in comparison to connections between individual and contextual factors (e.g. pride in citizenship), indicating a potential detachment from family resources as young people become more independent (Höltge et al., 2021).

Bridges between levels of protective factors are also likely to vary between cultures, as protective factors may be more or less important or available depending on context (Theron & Liebenberg, 2015; Ungar, 2008; Ungar et al., 2007). Indeed, in Höltge et al. (2021) study, interconnectivity between resources at an individual level and those related to caregivers declined as age increased in a sample of Canadian adolescents but formed a u-shaped pattern of influence for South African adolescents. Such variations may be explained by differences in the way adolescents interact with their social environments as they move towards independence. This study is part of an important element of resilience research, which asserts that there are culturally and contextually specific aspects of the lives of individuals that contribute to their positive adaptation (Ungar, 2008). Whether in contexts of structural and social disadvantage in high-income countries (Brody et al., 2013) or in countries with populations under-represented in the resilience literature (see e.g. Wu et al., 2014), there remains a lack of attention to the ethnocentrism in analysis of the protective factors that predict resilience.

Therefore, the aim of the present study was to develop our understanding of the interactivity of protective factors (within and across system domains) implicated in processes of resilience in emerging adults. The domains of protective factors we explored were psychological (i.e. personal skills and strengths) and social-ecological (resources in one’s environment), as protective factors from these domains are commonly identified and explored in studies of resilience, but seldom studied together (Fritz, De Graaff, et al., 2018). We also sought to address the lack of cross-cultural network studies by exploring the networks of individuals in diverse countries in order to understand how protective factor interactivity may vary, which can provide the foundations for research to develop contextually appropriate interventions to support the health and wellbeing of emerging adults during this critical period. In particular, we were guided by the following research questions:

1. How do the protective factor networks of individuals in different countries vary at overall structural and specific interrelation levels?
2. How do the bridge interrelations between different levels of protective factors (i.e. intra-person: personal skills and strengths and external: social-ecological resources) vary between these networks?

## Method

### Design

In the present study, we apply network modelling to a dataset collated by Edelman Intelligence. The original data collection was commissioned by Clear (a Unilever hair care brand) for a project that sought to explore individuals’ experiences of social anxiety, their levels of resilience and their functioning across various life domains. The dataset produced for the project was compiled from responses to an online survey engaged with individuals aged 16–29 in Brazil, China, Indonesia, Russia, Thailand, the US and Vietnam. These countries were selected by Clear, but their diversity provided a suitable sample to explore putative differences in the protective factors and their interrelations. The survey took place in November 2019 and participants were randomly recruited by three market research organisations (Dynata, Online Market Intelligence, and GMO Research) who drew on their nationally representative research panels (matched to available census data by sex, age and location in each country).

The participants had previously provided informed consent to take part in the survey and for their data to be anonymously used for research purposes. Dynata adheres to the Market Research Society code of conduct, and Online Market Intelligence and GMO Research adhere to the ESOMAR market research code of conduct. Secondary analyses of the dataset (the present study) were authorised by the lead author’s institutional Research Ethics Board. The study conforms to STROBE guidelines for cross-sectional research (Vandenbroucke et al., 2007) and the reporting standards for psychological network analyses (Burger et al., 2020).

### Participants

The dataset comprised responses from 7001 individuals aged 16–29 years. We selected a subset of young adults aged 20–29 (*n* = 5283), given suggestions that a broadly equivalent cross-country threshold for adulthood is being older than 19 (World Health Organization, 2013). Upon review, we found that 804 individuals could be excluded for selecting the same response for all items of the measures in the survey, as they were likely not properly engaged and may have been ‘straight-lining’ (see Johnson, 2016). This led to a final sample of 4479 individuals aged 20–29 (*M* = 24.53, *SD* = 2.86). Fifty-two percent of the sample identified as female and 0.71% did not identify as male or female (14 preferred not to declare, 13 identified as non-binary, and five preferred to self-describe) (Table 1).

**Table 1.** Sample characteristics and transformed scores on the resilience measures.

	**N (% Female)**	**Age**	**ARM-R**		**RRM**	
		***M (SD)***	***M (SD)***		***M (SD)***	
Brazil	651 (50.08)	24.49 (2.82)	63.26	(17.54)	61.95	(19.83)
China	708 (54.80)	24.40 (2.84)	59.10	(16.29)	58.25	(16.38)
Indonesia	576 (49.48)	24.20 (2.79)	63.40	(17.65)	63.20	(18.45)
Russia	673 (52.30)	25.16 (2.97)	61.76	(16.66)	62.33	(18.03)
Thailand	603 (51.08)	24.38 (2.86)	60.31	(16.54)	60.43	(17.25)
US	619 (56.06)	24.50 (2.75)	60.49	(17.44)	60.40	(16.78)
Vietnam	649 (53.00)	24.54 (2.92)	61.94	(17.34)	58.93	(18.05)
Total	4479 (52.47%)	24.53 (2.86)	61.41 (17.10)		60.73 (17.90)	

*Note*: Scores on the measures are transformed to be out of 100 for ease of comparison.

### Measures

We operationalise resilience as a multisystemic process involving the interaction of different levels of protective factors that facilitate positive outcomes despite adversity. The dataset we received contained responses to measures of such protective factors at two different system levels. These were the *Adult Resilience Measure-Revised* (ARM-R; Jefferies et al., 2018; Liebenberg & Moore, 2018), which concerns protective factors of a social-ecological nature, and the *Rugged Resilience Measure* (RRM; Jefferies et al., 2021), which concerns psychological protective factors.

The ARM-R is a 17-item measure of social-ecological resilience (score range = 17–85), where higher scores indicate a greater presence of (and engagement with) supportive resources in an individual’s environment, indicative of an individual’s resilience. These social-ecological resources include a sense of belonging in one’s community, family and peer support, and having opportunities to apply one’s abilities (see Table 2 for items). The measure was originally produced as part of an international resilience study where 14 communities across 11 countries contributed to the development of a version of the measure for children and youth (Resilience Research Centre, 2003). The measure was then adapted for adults and its psychometric properties confirmed (Antora, 2018; Liebenberg & Moore, 2018; Robinson, 2013). It is now widely used in diverse country contexts (see cyrm. resilienceresearch.org/properties). In this sample, the ARM-R had good internal consistency (α=.91; ω_h_=.78).

**Table 2.** Average item characteristics across countries.

**Label**	**Item**	***M (SD)***	**M_EI_**	**M_EIB_**
A1	Cooperation with others	3.46 (1.02)	.964	.140
A2	Valuing qualifications and skills	3.56 (1.04)	.796	.148
A3	Ability to adapt behaviour to fit a situation	3.40 (1.04)	.979	.277
A4	Family support	3.57 (1.13)	1.007	.052
A5	Family knowing a lot about the individual	3.45 (1.17)	.938	.076
A6	Availability of food	3.78 (1.04)	.718	.127
A7	Others enjoying one’s company	3.27 (1.05)	.902	.128
A8	Talking with family/partner about feelings	3.35 (1.18)	.850	.093
A9	Peer support	3.39 (1.11)	.894	.041
A10	Sense of belonging in community	3.22 (1.10)	.969	.045
A11	Ability to rely on family/partner during hardship	3.61 (1.11)	1.088	.129
A12	Ability to rely on friends during hardship	3.22 (1.12)	.867	.001
A13	Fair treatment in community	3.38 (1.04)	.855	.079
A14	Opportunities to demonstrate responsibility	3.53 (1.01)	.841	.202
A15	Feeling secure when with partner/family	3.71 (1.11)	.95	.023
A16	Opportunities to apply abilities (skills, a job, caring for others)	3.42 (1.04)	.972	.224
A17	Enjoying family’s/partner’s cultural and family traditions	3.47 (1.11)	.870	.073
R1	Self-belief	3.52 (1.11)	.947	.202
R2	Adapting to challenging situations	3.38 (1.03)	.963	.179
R3	Problem solving	3.39 (1.01)	.982	.175
R4	Perseverance	3.52 (1.03)	.916	.222
R5	Ability to cope with competing demands	3.27 (1.02)	.911	.176
R6	Optimism	3.52 (1.06)	.966	.104
R7	Emotional self-regulation	3.34 (1.11)	.582	.181
R8	Pride in achievements	3.55 (1.08)	.778	.286
R9	Willingness to take on challenges	3.39 (1.04)	.949	.089
R10	Meaning making	3.41 (1.11)	.977	.245

*Note*: Rx=RRM item; Ax =ARM item; M = average score; SD = standard deviation of average score; M_EI_ = Average expected influence; M_EIB_ = Average bridge expected influence.

The 10-item RRM is a measure of psychological resilience, broadly assessing the level of inner strengths and intrapersonal skills that individuals possess. It contains items linked to qualities that have been historically associated with individual resilience (e.g. perseverance, problem solving and optimism). The RRM also had good internal consistency in this sample (α=.87; ω_h_=.83).

The ARM-R and RRM complement each other as the ARM-R predominantly focuses on social-ecological protective resources in an individual’s environment while the RRM focuses on intrapersonal protective individual skills and strengths. In combination, the two measures provide a holistic appraisal of resilience (Jefferies et al., 2021). Importantly, the usage of both measures allows us to disentangle how protective factors from these different domains (social-ecological resources in the ARM-R and intrapersonal skills and strengths in the RRM) interact with each other to produce a cohesive system of protective factors.

Prior to the network modelling, we confirmed invariance of the measures across country contexts (see Supplemental Table S5). We also assessed conceptual overlap between the items of the measures using the *goldbricker* function of the *networktools* package (Hittner method; Jones, 2020). This analysis identifies strongly correlated item pairs (*r* ≥ .7) which have less than 20% unique correlations with other items (Everaert & Joormann, 2019), as such potential repetition can skew networks (Fried & Cramer, 2017). No topological overlap was detected.

### Procedure

All analyses were conducted using R v4.0.0 (Arbor Day) via RStudio v1.2.5042 (R Core Team, 2020; RStudio Team, 2020). A full list of the packages used in the study is provided in the supplements. A missing data analysis was not required, as participants had been required to respond to every item of each measure in order to complete the survey.

### Network estimation

When presented graphically, network models consist of nodes (circles) and edges (lines). The nodes in the models we estimated represent the items of the ARM-R and RRM. Hence there were 27 in each country network. The edges are the connections between the nodes, where thicker lines represent stronger interrelationships between variables. Edges may go between nodes of the same group (e.g. between ARM-R nodes) as well as between nodes of different groups (i.e. between ARM-R and RRM nodes). The latter are known as ‘bridge edges’ (Jones et al., 2019) and are the focus of this study.

Nodes in the networks were initially arranged using the Fruchterman and Reingold (1991) algorithm which places strongly correlated nodes together, but the *averageLayout function* of the *qgraph* plotting package was employed to reposition the nodes so they would be organised in the same way for all networks to facilitate visual comparison (Fritz, Fried, et al., 2018). As such, the distance between the nodes should not be interpreted (Jones et al., 2018).

Network models can be constructed using zero-order or partial correlations between variables. However, as all nodes will be associated to some degree, this leads to dense networks with a noteworthy risk of false-positive associations and where the most important connections or differences between networks can be hard to discern (Costantini et al., 2015; Epskamp & Fried, 2018). To avoid this problem, several methods have been proposed to reduce the number of edges, the most popular of which is the graphical least absolute shrinkage and selection operator (glasso), which uses a tuning parameter to limit the sum of absolute partial correlation coefficients, causing all estimates to shrink and some to become zero, thereby creating sparser networks (Costantini et al., 2015; Epskamp & Fried, 2018; Friedman et al., 2008). However, regularisation techniques like glasso have recently come under scrutiny (see Williams & Rast, 2020) and alternatives have demonstrated a lower false-positive rate and greater generalisability (Williams et al., 2019). We used thresholding as a non-regularisation estimation technique to achieve sparser networks, using the *prune* function of the package *psychonetrics*. This process removes edges from a saturated partial correlation matrix (estimated by *ggm*) which are not significant at a given level of *α* (.05 in this study) (Epskamp, 2021; Epskamp et al., 2020; Kan et al., 2020). The process is recursive and, therefore, a model is re-evaluated after initial pruning to determine whether further edges should be removed. Fit indices are provided to evaluate the goodness of the pruned model (see Supplemental Table S1).

To ensure the estimated networks were robust, we performed 1000 case-drop bootstraps (Epskamp et al., 2018) where 25% of the data were omitted and the models re-estimated using the same process as above (Epskamp, 2020). Omitting a quarter of the sample has been suggested as an appropriate amount of cases to drop to determine the stability of network structures (Epskamp, 2020).

### Contrasting the networks

To formally compare the network models, Bayesian posterior predictive check tests were used to determine whether pairs of country networks significantly differed at an overall structural level. This global test used the *BGGM* package (Williams et al., 2020). We then contrasted the networks on their global expected influence and global bridge expected influence (see later) as more specific tests of potential country variation, using the *NetworkComparisonTest* (n_permutations_ = 5000) (van Borkulo et al., 2017).

We also determined the number of significantly different bridge edges and non-bridge edges between pairs of country networks using the *NetworkComparisonTest*, as this indicates whether differences between the networks are due to differences within or between protective factor domains in the system. A similar process also revealed the number of nodes that significantly differed between networks in terms of their expected influence and their bridge expected influence. Moreover, we produced variability networks based on the standard deviation of the edges and the expected influence of the nodes. These variability networks provide an insight into where the most similarities and differences may be found (Fried et al., 2018; Höltge et al., 2020).

Finally, we briefly reviewed the node centrality coefficients across countries (Costantini et al., 2015). These are indices of interrelatedness among nodes that help to interpret the models. In particular, we assessed expected influence, which is the relative sum of all interrelation values of a node with the nodes it is directly related to. The expected influence coefficient indicates whether a node has an activating or deactivating role in the network, depending on whether it has more positive or negative connections. It is useful for studies in the social sciences where relationships between variables can be negative and therefore where an absolute sum (node strength) would be less appropriate (Robinaugh et al., 2016). Furthermore, the node(s) with the highest expected influence may be considered particularly relevant in the networks (Hevey, 2018; Stochl et al., 2019).

We obtained expected influence coefficients for each node per country network, as well as the global expected influence of a network, which would indicate overall relative node interconnectivity in the country networks. We then did the same within each group of nodes (the ARM and RRM groups) to determine connectivity within each of the resilience system domains for the different country networks.

Additionally, we sought to determine bridge centrality indices, which indicate the connectivity of a single node of one group of items (e.g. the RRM items) with the connected nodes of the other group of items (i.e. the ARM items). Node bridge indices were normalised to address the unequal number of nodes between the RRM and the ARM (Jones et al., 2019), and were derived using the *networktools* function. Global bridge expected influence was also derived to gauge the overall interconnectivity of two resilience system domains (i.e. RRM and ARM).

## Results

**Figure 1.**
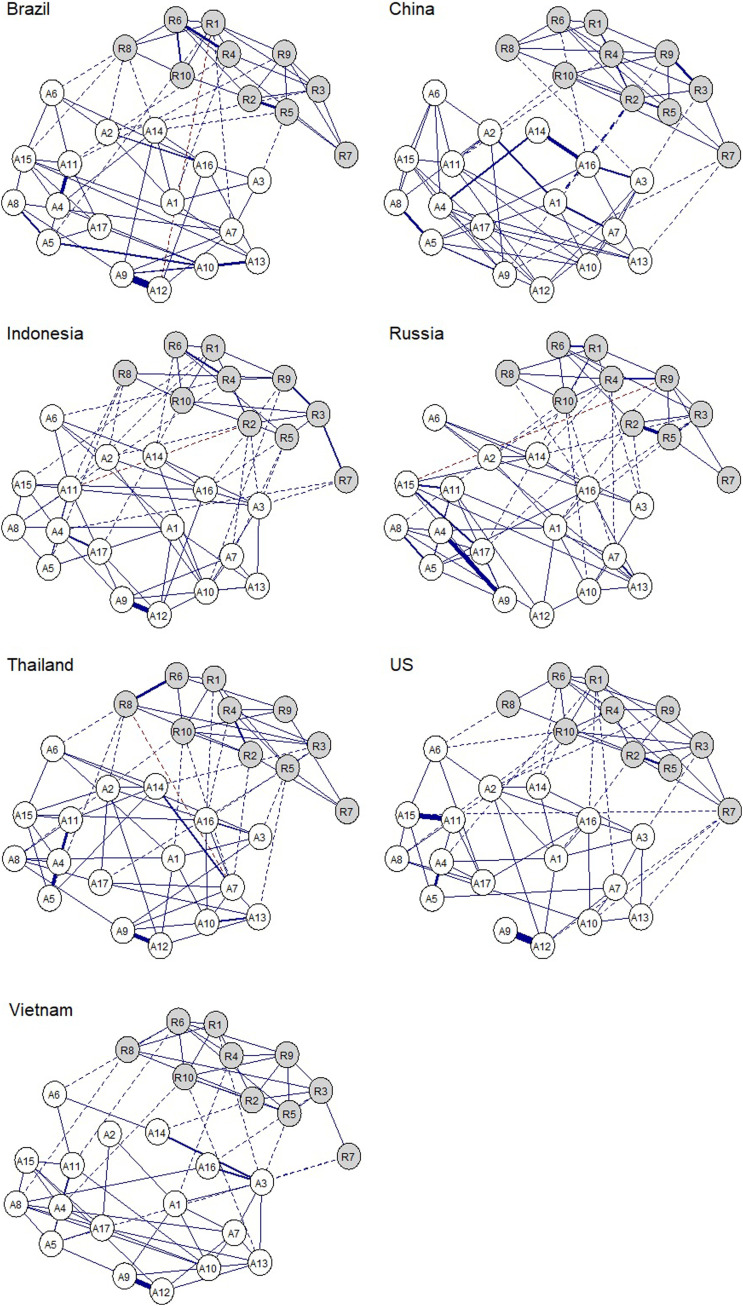
Pruned country partial correlation network models. *Note:* Grey nodes = RRM; White nodes = ARM; see Table 2 for node labels; blue solid lines = within-system positive edges; blue dashed lines = positive bridge edges; red dashed lines = negative bridge edges. The arrangement of nodes is an average across all networks for clarity.

The fit of the estimated models was good (BIC=39,397.69–50,092.55; RMSEA=.03–.05; CFI=.92–.98; TLI=.90–.97; Supplemental Table S1). The case-dropping bootstraps indicated that the edges present in the estimated networks (Supplemental Table S2; Figure 1) were also present in about three-quarters of bootstraps (72.11%; Supplemental Table S3).

The results of the Bayesian posterior predictive check test indicated that each of the country networks significantly differed from each other (Kullback–Leibler divergence = 1.46–2.22, *p*s < .001), suggesting that the overall structure of the protective factor networks was distinguishable by country (Supplemental Table 4). Figure 1 illustrates this, where differences can be seen in the presence and magnitude of edges both within and between the system domains.

Variation between the networks was also reflected in the edge-difference tests, which ranged between a minimum of 13 significantly different interrelations between the nodes of the Indonesian and Vietnamese networks to 28 when contrasting Indonesian and Russian networks (*M* = 21.24 edges, *SD* = 5.75; *p*s < .05; Table 3). An unexpected finding lies in the negative interrelations that were detected in four of the country networks (Figure 1). These negative edges were only found between the protective factor groups (only bridge edges; reported further in the next section).

**Table 3.** Number of significantly different edges (bridges and non-bridges) and expected influence coefficients of nodes (bridge expected influence and overall expected influence) between countries (*p* < .05).

	China		Indonesia			Russia			Thailand			US			Vietnam		
	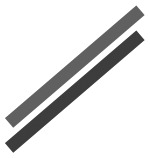	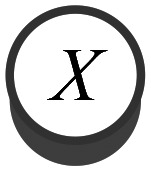	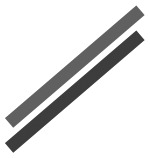		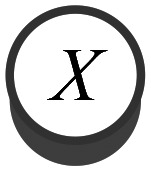	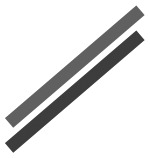		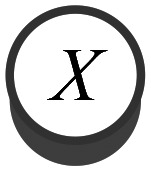	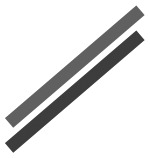		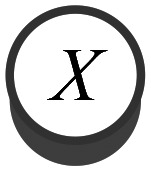	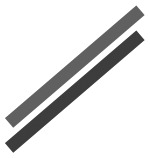		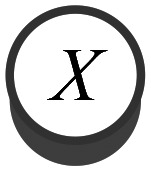	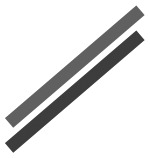		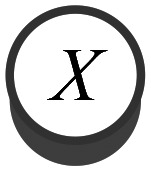
Brazil	7 (28)	4 (4)	7 (20)	2 (3)		5 (27)	6 (6)		4 (24)	3 (2)		3 (15)	4 (5)		5 (16)	3 (5)	
China			7 (19)	2 (1)		10 (27)	3 (4)		5 (27)	3 (1)		4 (19)	2 (1)		7 (24)	0 (2)	
Indonesia						10 (28)	6 (1)		7 (18)	0 (1)		10 (18)	1 (3)		8 (13)	0 (1)	
Russia									7 (31)	1 (3)		8 (25)	4 (7)		6 (25)	2 (4)	
Thailand												1 (12)	2 (2)		3 (14)	0 (3)	
US															7 (16)	1 (3)	

*Note*: Line = edges; Circle = nodes; Numbers in brackets indicate how many interrelations or nodes (in terms of expected influence) significantly differed between two countries (e.g., the magnitude of 16 interrelations and the expected influence of five nodes significantly differed between the networks of Brazil and Vietnam). Numbers without brackets indicate how many interrelations between domains significantly differed between two countries and the number of significantly differing nodes in terms of their bridge expected influence (e.g., five of the interrelations between RRM and ARM nodes significantly differed between the networks of Brazil and Vietnam, as well as three nodes which significantly differed in terms of their bridge expected influence).

Table 4 provides further insight into overall differences through the results of the global expected influence tests (EI_G_). A third of the network pairs significantly differed in terms of their global expected influence (*p*s < .05). The largest difference was detected between Indonesia (EI_G_ = 12.51) and Vietnam (EI_G_ = 11.89), while the most similar were China (EI_G_ = 12.09) and the US (EI_G_ = 12.08) (see Table 5).

**Table 4.** Global expected influence tests.

	China		Indonesia		Russia		Thailand		US		Vietnam	
	EI_G_	EI_BG_	EI_G_	EI_BG_	EI_G_	EI_BG_	EI_G_	EI_BG_	EI_G_	EI_BG_	EI_G_	EI_BG_
Brazil	.295 (.035)*	.027 (.938)	.129 (.338)	.741 (.053)	.221 (.079)	.185 (.549)	.002 (.989)	.273 (.417)	.298 (.056)	.572 (.127)	.487 (.002)*	.164 (.630)
China			.425 (.021)*	.769 (.078)	.075 (.624)	.212 (.571)	.297 (.122)	.300 (.483)	.002 (.991)	.600 (.189)	.191 (.359)	.190 (.655)
Indonesia					.350 (.027)*	.557 (.130)	.127 (.405)	.468 (.267)	.427 (.012)*	.172 (.697)	.616 (.005)*	.580 (.163)
Russia							.223 (.134)	.089 (.812)	.077 (.617)	.303 (.388)	.266 (.122)	.022 (.953)
Thailand									.300 (.063)	.300 (.488)	.489 (.012)*	.110 (.790)
US											.189 (.312)	.410 (.332)

*Note*: *p*-values in brackets. EI_G_ = Global expected influence; EI_BG_ = Global bridge expected influence. *p<.05.

**Table 5.** Expected influence estimates of nodes per group (including bridges), within groups (excluding bridges), between groups (bridges), and global expected influence.

	Total group		Within group (excl. bridge)		Between group (bridge)	Global
	RRM	ARM	RRM	ARM	–	–
Brazil	4.68 (.94)	7.70 (.91)	3.89 (.08)	6.90 (.05)	1.59	12.38
China	4.32 (.86)	7.77 (.91)	3.54 (.07)	6.99 (.05)	1.56	12.09
Indonesia	4.59 (.92)	7.92 (.93)	3.43 (.07)	6.76 (.05)	2.33	12.51
Russia	4.53 (.91)	7.64 (.90)	3.64 (.07)	6.75 (.05)	1.77	12.16
Thailand	4.37 (.87)	8.01 (.94)	3.44 (.07)	7.08 (.05)	1.86	12.38
US	4.55 (.91)	7.53 (.89)	2.47 (.07)	6.45 (.05)	2.16	12.08
Vietnam	4.36 (.87)	7.54 (.89)	3.48 (.07)	6.66 (.05)	1.75	11.89

*Note*: Average estimates are given in brackets, given the uneven number of nodes per group. EI_NB_=expected influence (excluding bridges); EI_G_=global expected influence; EI_B_=global bridge expected influence.

**Figure 2.**
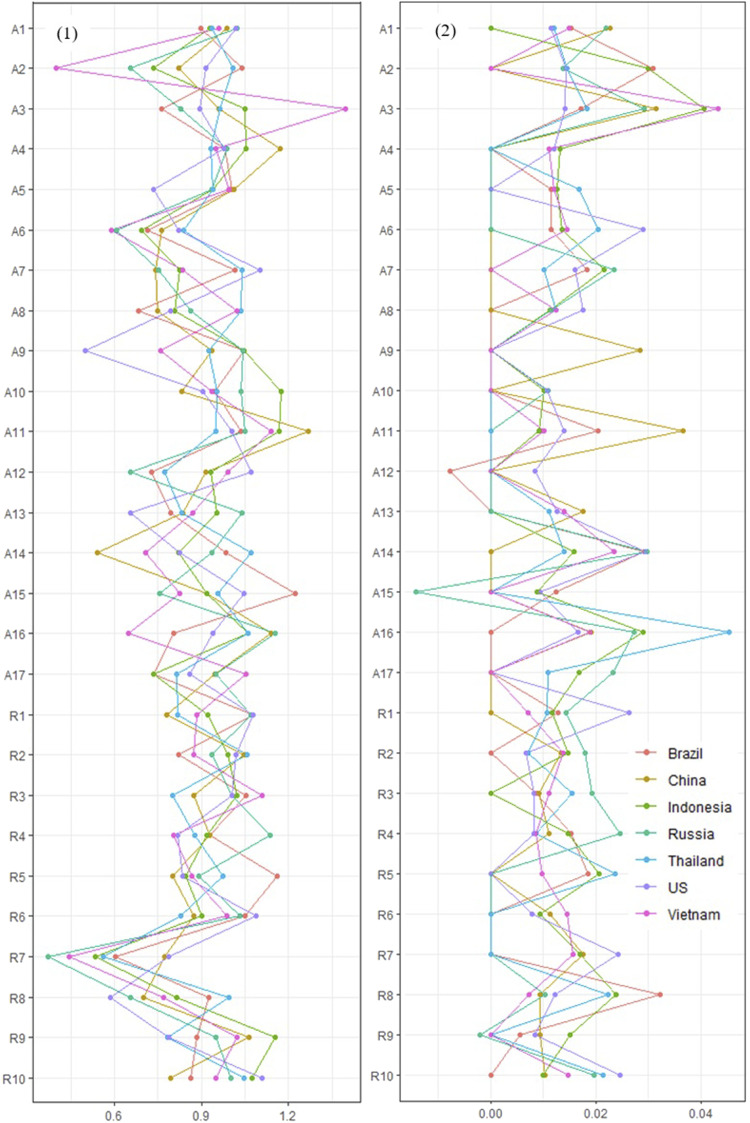
Node expected influence (1) and bridge expected influence (2). *Note:* Rx=RRM item; Ax=ARM item. Bridge expected influence values are normalised due to the unequal number of nodes per group (see method section).

In terms of their overall expected influence, the number of nodes significantly differing between the country networks ranged from 1–7 (*M* = 2.95, *SD* = 1.77). The average expected influence of nodes across the country networks can be found in Table 2. The nodes with the highest expected influence were A4 (family support; M_EI_ = 1.01) and A11 (family/partner standing by during difficult times, M_EI_ = 1.09). However, these were typically not the most relevant nodes when considering individual country networks, which varied between each network (Figure 2, panel 1).

**Figure 3.**
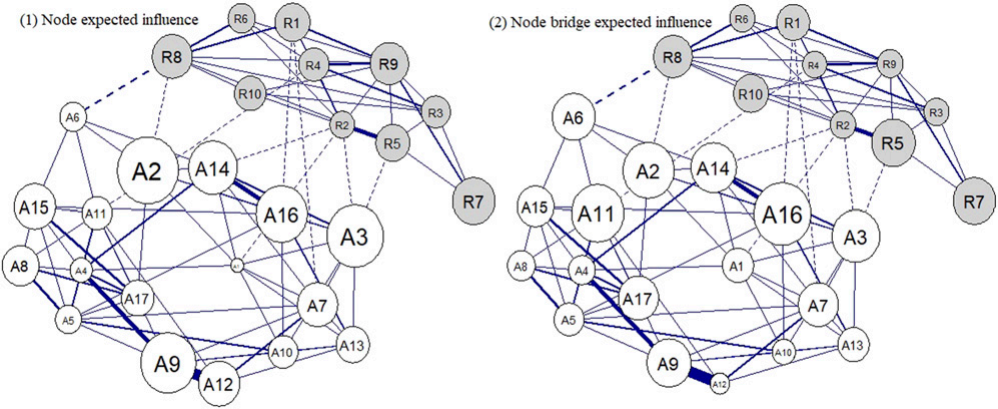
Cross-country variability network. *Note:* Edge thickness reflects the standard deviation of the edge weights across all countries (the same in both plots). Node size reflects the standard deviation of the node expected influence centralities across all countries. The thicker the edge/larger the node, the higher its variation across countries. Top 25% of varying edges displayed. Solid line = non-bridge edge; Dashed = bridge edge.

The cross-country variability depicted in the plots of Figure 3 provides an overview of further common areas of variation between the networks. The greatest variation in node interrelations was found between ARM nodes general peer support (A9) and peer support during hardship (A12). This was a strong connection in most of the networks but was notably absent in the networks of China and Russia.

No significant differences were found between any of the networks in terms of their global bridge expected influence (Table 4; EI_BG_). However, the number of nodes that significantly differed in terms of their overall bridge expected influence ranged from 0–6 (*M* = 2.33, *SD* = 1.80). Furthermore, the number of significant bridge edges also varied from as low as one (between the US and Thailand networks) to 10 (between Russia and China, Russia and Indonesia, and Indonesia and the US) (*M* = 6.24, *SD* = 2.41 bridge edges). Therefore, while the overall bridge connectivity remained similar between the country networks, the bridges themselves varied (see Table 2 and Figure 2 panel 2).

Figure 2 panel 1 also illustrates that there was greater variability in the expected influence of the social-ecological resources (i.e. the ARM nodes) compared to the intrapersonal skills and strengths (i.e. the RRM nodes), a finding consistent across the country networks (but not as apparent in bridge expected influence). A further pattern was that R7 (emotional self-regulation) appeared independent across many of the country networks. The largest variation in the bridges between the protective factor groups was between the ability to access food (A6) and pride in achievements (R8), which was present in most networks aside from those of China and Russia, and also Indonesia.

In terms of the negative interrelations between protective factor groups (bridge edges) in the Brazil network, this was between self-belief (R1) and the ability to rely on friends during hardship (A12), while in Indonesia, a negative interrelation was found between adapting to challenges (R2) and the ability to rely on a partner/family during hardship (A11). In Russia, it was between the ability to take on challenges (R9) and security from a partner/family (A15), and in Thailand, it was between pride in achievements (R8) and others enjoying one’s company (A7). Figure 2 panel 2 reflects some of these findings, where a number of the nodes had overall negative bridge expected influence, indicating a potentially antagonistic relationship between protective factor domains in two of the networks: Brazil (peer support during hardship, A12) and Russia (security from partner/family, A15; and ability to take on challenges, R9).

## Discussion

To our knowledge, this is the first study to report a cross-cultural network analysis of resilience among emerging adults. Our findings present patterns of interconnectivity within and between different system domains of protective factors which foreground similarities and differences between emerging adults of different country contexts. In particular, we found that all country networks differed from each other at overall structural levels and when examining specific protective factors and their interrelations with others. However, differences were not as prevalent when examining overall centrality estimates, such as global expected influence or global bridge expected influence. This suggests that there may be an overall level of connectivity within and between resilience system domains which is similar across countries, but that the specific inter- and intra-relations that comprise this connectivity among protective factors will vary. For instance, emotional self-regulation demonstrated high variability between countries in terms of its connections with other psychological skills and strengths as well as the social-ecological resources. In the networks for Brazil, China, Thailand and the US, it was connected with various other skills and strengths, but in the networks of Indonesia and Vietnam it only shared a single significant connection with the strength of problem solving (other strengths were more strongly connected in these networks). This same protective factor was variously disconnected from the social-ecological resources in the networks of Brazil, Russia and Thailand, but was well-connected with resources in the US network.

The varying independence and association of protective factors between countries supports arguments of cultural heterogeneity patterns of resilience (Panter-Brick, 2015; Ungar, 2013, 2015) and may be partially accounted for by country-specific norms. For instance, feeling supported by friends was independent of most protective factors in the US sample, but in the Brazilian sample, it was related to knowing how to behave in different social situations, and in the Vietnam sample it was related to social cooperation. In cultures where social cohesion is emphasised and individuality deemphasised, support from others may be more conditional upon one’s social competence (Demir et al., 2012; Mortenson, 2009). Likewise, one of the stronger interconnections among many of the country networks was between peer support and an ability to rely on friends during hardship, though this was comparatively weaker for the networks of China and Russia, where the notion of peers and friends may overlap to a lesser degree (Doucerain et al., 2021; Rubin et al., 2010). A further difference can be observed between the protective factors concerning adapting to challenging situations and cooperation with others, which was a relatively strong bridge connection in the network of China, yet weaker or absent in the other country networks, which may reflect the importance of social elements in facilitating adaptation to challenges in China (e.g. potentially related to ‘guanxi’; Warren et al., 2004). Although norms may therefore account for variations in the differences between interrelations among social-ecological protective factors, and sometimes the bridges between these and other systems, it is harder to account for the variation in the interrelationships of psychological strengths between countries, such as self-belief and perseverance, which were associated in the networks of Brazil, China, Vietnam, but not in those of Indonesia, Russia, Thailand and the US. A focused investigation of cultural features related to each of the protective factors may help to further our understanding of the variability in their interplay.

A further interesting finding was the detection of significant negative interrelations, as each of the factors are thought to be protective, and therefore a positive manifold (all positive interrelations) may be expected (Borsboom et al., 2011; Horn & Cattell, 1966). Williams (2020) has suggested that some negative edges may be expected in networks with a larger number of nodes, especially involving ordinal data, as these may be false positives. However, the pruning process involved in the estimation of the study networks aimed to minimise the rate of false positives, and the bootstraps indicated that these particular edges were present in more than half of the case-drop resampled models, suggesting they may be true effects (Supplemental Table S3). These negative edges were only found between protective factors of different domains, where a negative association may be more plausible. For instance, in the network of the Russian sample, greater security provided by family or partners was associated with lower perceived ability to take on challenges. Individuals who experience higher levels of security from those close to them may have a lower sense of personal self-efficacy, and vice versa. Similarly, in the Thai network, others valuing one’s company was associated with a lower sense of pride in one’s achievements. The importance of social harmony and cohesion in Thailand (Cheung et al., 2014; Niffenegger et al., 2006) suggests that interpersonal relationships take precedence over one’s own standing (Knutson et al., 2003), and so although recognising and embracing one’s successes is generally thought to be important for resilience (Connor & Davidson, 2003; Rutter, 1985), in some cultural contexts this may be less consistent with social norms, where celebrating group successes is more appropriate.

The findings of this study expand our understanding of emerging adult resilience by illuminating the variable nature of interrelationships within and between system levels of protective factors and how these also vary between countries. In particular, while the identification of some within-system protective factor relationships echoes previous findings (e.g. Fritz, Fried, et al., 2018), the relationships detected between protective factors of different systems (i.e. between particular skills and strengths and social-ecological resources) furthers our understanding of their connectivity, as does the finding that the overall level of this connectivity was similar between country contexts, but that particular connections varied. This latter point deserves further translational research, as the findings may be important for practitioners. For instance, knowing that a factor such as perseverance was generally well-connected with other strengths and skills and also with social-ecological resources could encourage intervention developers to focus on this in resilience-building initiatives, as this suggests it may play a more influential role than other factors. However, recognising that the most connected factors tend to vary by country context indicates that what might be most beneficial to focus on in one location may differ from another.

These findings are an important step in furthering our understanding of multisystemic resilience and for identifying areas of focus for culturally nuanced resilience-building interventions that may help to address the disproportionate prevalence of mental health issues in emerging adults (Arnett et al., 2014). However, it is critical that further study take place prior to application (Bringmann et al., 2019; Dablander & Hinne, 2019). In particular, longitudinal studies would be a suitable next step to confirm that changes to certain protective factors do indeed lead to changes in others they have been associated with.

A further implication is that although positive interconnections may generally be anticipated between protective factors within and between system domains, the negative interrelations we detected suggests that the presence or strength of factors at one level may be associated with an absence or less of another at a different system level. For instance, efforts to bolster such factors (e.g. in Brazil: relying on friends during hardship) may be achieved at the expense of something else (greater self-belief).

### Limitations and future directions

This study draws on cross-sectional data to provide an overview of patterns of protective factors among adults from different countries. This led to networks with bidirectional edges where associations are visible but conclusions about the directionality between protective factors are inhibited: for example, in the Russian sample, it is unclear whether (i) perceiving lower levels of security from one’s partner or family pushes individuals to gain a greater sense of personal efficacy, (ii) lacking such a sense of efficacy prompts individuals to seek support from others, or whether (iii) there is some reciprocity between these protective processes. Future longitudinal studies could shed light on directional effects.

Additionally, we acknowledge that network models may be susceptible to measurement error (Wang et al., 2012), potentially manifesting in biased edges in the networks. A latent variable approach could cater to such sources of error (Borsboom et al., 2003; Muthén, 2002), and there are promising methods combining latent and network approaches (e.g. Residual Network Modelling; Epskamp et al., 2017), although some epistemological issues are still being debated (Bringmann & Eronen, 2018; Guyon et al., 2017; Rhemtulla et al., 2020).

A further important avenue for research would be to explore the connectivity of protective factor networks in samples of individuals who have experienced different forms of adversity. In the present study, in the absence of known adversity, the findings inform us of the ‘natural’ interrelations of previously established protective factors, and if an adverse event were to affect a particular resource (e.g. family support), the patterns of connectivity presented in our findings suggest which other factors may be impacted (Fonseca-Pedrero, 2017). That said, an analysis of those experiencing a specific form of adversity could reveal the extent to which combinations of protective factors are important, mutually supportive or inhibitory in such adversity contexts (Kalisch et al., 2019; Lunansky et al., 2021), in addition to how these patterns may or may not vary cross-culturally.

This study involved protective factors of a psychological and social-ecological nature. Although inclusion of further protective factors from these domains which were not included in the representative measures in this study (e.g. cognitive reappraisal and self-esteem; Fritz, De Graaff, et al., 2018) may not be expected to lead to different outcomes, an important extension could include protective factors from additional domains such as the built and natural environment (Ungar, 2021; Ungar & Theron, 2020; van den Bosch & Ode Sang, 2017; Zhang et al., 2018). Such extensions would give further depth to multisystemic understandings of resilience.

Furthermore, researchers who work with older cohorts have advocated for different approaches to resilience for older adults (Cosco et al., 2019; Ong et al., 2009). It would therefore be important to extend this work to older groups of adults to determine whether protective factor interrelations are invariant across age.

## Conclusion

In their recent review of network studies in psychopathology, Robinaugh et al. (2020) noted that it may be prudent to consider that we are at an early stage of phenomena detection. Researchers are undertaking exploratory network analyses to uncover patterns in their data, and such foundational work is necessary prior to the development of formal theories and, in turn, intervention development. In this study, we contribute to the foundations of resilience network analyses by reporting exploratory findings that show heterogeneity in adult resilience networks across different country contexts. In particular, interrelations between resilience protective factors vary, where supportive connections may be found within and between different system domains of protective factors, while potentially inhibiting connections also occur between protective factors of different system domains.

## Supplemental Material

sj-pdf-1-eax-10.1177_21676968221090039 – Supplemental Material for A Cross-Country Network Analysis of Resilience Systems in Young Adults Click here for additional data file. Supplemental Material, sj-pdf-1-eax-10.1177_21676968221090039 for A Cross-Country Network Analysis of Resilience Systems in Young Adults by Philip Jefferies, Jan Höltge, Jessica Fritz and Michael Ungar in Emerging Adulthood

## References

[bibr1-21676968221090039] Afifi T. O. MacMillan H. L. (2011). Resilience following child maltreatment: A review of protective factors. The Canadian Journal of Psychiatry, 56(5), 266–272. 10.1177/070674371105600505 21586192

[bibr2-21676968221090039] Antora S. (2018). The influence of visibility on mental health amongst the muslim female population in the United States. City University of New York. https://academicworks.cuny.edu/hc_sas_etds/291

[bibr3-21676968221090039] Arnett J. J. (2000). Emerging adulthood. A theory of development from the late teens through the twenties. The American Psychologist, 55(5), 469–480. 10.1037/0003-066x.55.5.469 10842426

[bibr4-21676968221090039] Arnett J. J. (2007). Emerging adulthood: What is it, and what is it good for? Child Development Perspectives, 1(2), 68–73. 10.1111/j.1750-8606.2007.00016.x

[bibr5-21676968221090039] Arnett J. J. Kloep M. Hendry L. B. Tanner J. L. (2010). Debating emerging adulthood: Stage or process. Oxford University Press.

[bibr6-21676968221090039] Arnett J. J. Žukauskienė R. Sugimura K. (2014). The new life stage of emerging adulthood at ages 18–29 years: Implications for mental health. The Lancet Psychiatry, 1(7), 569–576. 10.1016/S2215-0366(14)00080-7 26361316

[bibr7-21676968221090039] Benzies K. Mychasiuk R. (2009). Fostering family resiliency: A review of the key protective factors. Child & Family Social Work, 14(1), 103–114. 10.1111/j.1365-2206.2008.00586.x

[bibr8-21676968221090039] Block J. H. Block J. (1980). The role of ego-control and ego-resiliency in the organization of behavior. In Collins W. A. (Ed.), Development of cognition, affect, and social relations (Vol. 13, pp. 39–102). Psychology Press.

[bibr9-21676968221090039] Bonanno G. A. Diminich E. D. (2013). Annual research review: Positive adjustment to adversity -trajectories of minimal-impact resilience and emergent resilience. Journal of Child Psychology and Psychiatry, and Allied Disciplines, 54(4), 378–401. 10.1111/jcpp.12021 23215790 PMC3606676

[bibr10-21676968221090039] Bonanno G. A. Romero S. A. Klein S. I. (2015). The temporal elements of psychological resilience: An integrative framework for the study of individuals, families, and communities. Psychological Inquiry, 26(2), 139–169. 10.1080/1047840x.2015.992677

[bibr11-21676968221090039] Bonanno G. A. Westphal M. Mancini A. D. (2011). Resilience to loss and potential trauma. Annual Review of Clinical Psychology, 7(1), 511–535. 10.1146/annurev-clinpsy-032210-104526 21091190

[bibr12-21676968221090039] Borsboom D. (2017). A network theory of mental disorders. World Psychiatry: Official Journal of the World Psychiatric Association (WPA), 16(1), 5–13. 10.1002/wps.20375 28127906 PMC5269502

[bibr13-21676968221090039] Borsboom D. Cramer A. O. J. (2013). Network analysis: An integrative approach to the structure of psychopathology. Annual Review of Clinical Psychology, 9(1), 91–121. 10.1146/annurev-clinpsy-050212-185608 23537483

[bibr14-21676968221090039] Borsboom D. Cramer A. O. J. Schmittmann V. D. Epskamp S. Waldorp L. J. (2011). The small world of psychopathology. PLoS One, 6(11), e27407. 10.1371/journal.pone.0027407 22114671 PMC3219664

[bibr15-21676968221090039] Borsboom D. Mellenbergh G. J. Van Heerden J. (2003). The theoretical status of latent variables. Psychological Review, 110(2), 203–219. 10.1037/0033-295X.110.2.203 12747522

[bibr16-21676968221090039] Briganti G. Linkowski P. (2019). Item and domain network structures of the resilience scale for adults in 675 university students. Epidemiology and Psychiatric Sciences, 29, e33. 10.1017/S2045796019000222. 31006419 PMC8061136

[bibr17-21676968221090039] Bringmann L. F. Elmer T. Epskamp S. Krause R. W. Schoch D. Wichers M. (2019). What do centrality measures measure in psychological networks? Journal of Abnormal Psychology, 128(8), 892–903. 10.1037/abn0000446 31318245

[bibr18-21676968221090039] Bringmann L. F. Eronen M. I. (2018). Don’t blame the model: Reconsidering the network approach to psychopathology. Psychological Review, 125(4), 606–615. 10.1037/rev0000108 29952625

[bibr19-21676968221090039] Brody G. H. Yu T. Chen E. Miller G. E. Kogan S. M. Beach S. R. H. (2013). Is resilience only skin deep?: Rural African Americans’ socioeconomic status-related risk and competence in preadolescence and psychological adjustment and allostatic load at age 19. Psychological Science, 24(7), 1285–1293. 10.1177/0956797612471954 23722980 PMC3713113

[bibr20-21676968221090039] Brown K. Westaway E. (2011). Agency, capacity, and resilience to environmental change: Lessons from human development, well-being, and disasters. Annual Review of Environment and Resources, 36(1), 321–342. 10.1146/annurev-environ-052610-092905

[bibr21-21676968221090039] Burger J. Isvoranu A.-M. Lunansky G. Haslbeck J. Epskamp S. Hoekstra R. H. A. (2020). Reporting standards for psychological network analyses in cross-sectional data. PsyArXiv. 10.31234/osf.io/4y9nz 35404629

[bibr22-21676968221090039] Burt K. B. Paysnick A. A. (2012). Resilience in the transition to adulthood. Development and Psychopathology, 24(2), 493–505. 10.1017/S0954579412000119 22559126

[bibr23-21676968221090039] Cheung C. Chan R. K. Ho W. (2014). Feeling close to fellow citizens in Hong Kong, Korea, Taiwan, and Thailand. Social Indicators Research, 119(1), 25–48. 10.1007/s11205-013-0483-8

[bibr24-21676968221090039] Chmitorz A. Kunzler A. Helmreich I. Tüscher O. Kalisch R. Kubiak T. Wessa M. Lieb K. (2018). Intervention studies to foster resilience—a systematic review and proposal for a resilience framework in future intervention studies. Clinical Psychology Review, 59, 78–100. 10.1016/j.cpr.2017.11.002 29167029

[bibr25-21676968221090039] Connor K. M. Davidson J. R. T. (2003). Development of a new resilience scale: The connor-davidson resilience scale (CD-RISC). Depression and Anxiety, 18(2), 76–82. 10.1002/da.10113 12964174

[bibr26-21676968221090039] Contreras A. Nieto I. Valiente C. Espinosa R. Vazquez C. (2019). The study of psychopathology from the network analysis perspective: A systematic review. Psychotherapy and Psychosomatics, 88(2), 71–83. 10.1159/000497425 30889609

[bibr27-21676968221090039] Cosco T. D. Kok A. Wister A. Howse K. (2019). Conceptualising and operationalising resilience in older adults. Health Psychology and Behavioral Medicine, 7(1), 90–104. 10.1080/21642850.2019.1593845 34040841 PMC8114384

[bibr28-21676968221090039] Costantini G. Epskamp S. Borsboom D. Perugini M. Mõttus R. Waldorp L. J. (2015). State of the aRt personality research: A tutorial on network analysis of personality data in R. Journal of Research in Personality, 54, 13–29. 10.1016/j.jrp.2014.07.003

[bibr29-21676968221090039] Dablander F. Hinne M. (2019). Node centrality measures are a poor substitute for causal inference. Scientific Reports, 9(1), 6846. 10.1038/s41598-019-43033-9 31048731 PMC6497646

[bibr30-21676968221090039] Demir M. Jaafar J. Bilyk N. Ariff M. R. M. (2012). Social skills, friendship and happiness: A cross-cultural investigation. The Journal of Social Psychology, 152(3), 379–385. 10.1080/00224545.2011.591451 22558831

[bibr31-21676968221090039] Doucerain M. M. Ryder A. G. Amiot C. E. (2021). What are friends for in Russia versus Canada?: An approach for documenting cross-cultural differences. Cross-Cultural Research, 55(4), 382–409. 10.1177/10693971211024599

[bibr32-21676968221090039] Egeland B. Carlson E. Sroufe L. A. (1993). Resilience as process. Development and Psychopathology, 5(4), 517–528. 10.1017/S0954579400006131

[bibr33-21676968221090039] Epskamp S. (2020). Psychometric network models from time-series and panel data. Psychometrika, 85(1), 206–231. 10.1007/s11336-020-09697-3 32162233 PMC7186258

[bibr34-21676968221090039] Epskamp S. (2021). Psychonetrics readme. https://github.com/SachaEpskamp/psychonetrics

[bibr35-21676968221090039] Epskamp S. Borsboom D. Fried E. I. (2018). Estimating psychological networks and their accuracy: A tutorial paper. Behavior Research Methods, 50(1), 195–212. 10.3758/s13428-017-0862-1 28342071 PMC5809547

[bibr36-21676968221090039] Epskamp S. Fried E. I. (2018). A tutorial on regularized partial correlation networks. Psychological Methods, 23(4), 617–634. 10.1037/met0000167 29595293

[bibr37-21676968221090039] Epskamp S. Isvoranu A.-M. Cheung M. (2020). Meta-analytic Gaussian network aggregation. PsyArXiv 10.31234/osf.io/236w8 PMC9021114 34264449

[bibr38-21676968221090039] Epskamp S. Rhemtulla M. Borsboom D. (2017). Generalized network psychometrics: Combining network and latent variable models. Psychometrika, 82(4), 904–927. 10.1007/s11336-017-9557-x 28290111

[bibr39-21676968221090039] Everaert J. Joormann J. (2019). Emotion regulation difficulties related to depression and anxiety: A network approach to model relations among symptoms, positive reappraisal, and repetitive negative thinking. Clinical Psychological Science, 7(6), 1304–1318. 10.1177/2167702619859342

[bibr40-21676968221090039] Fergus S. Zimmerman M. A. (2005). Adolescent resilience: A framework for understanding healthy development in the face of risk. Annual Review of Public Health, 26(1), 399–419. 10.1146/annurev.publhealth.26.021304.144357 15760295

[bibr41-21676968221090039] Fonseca-Pedrero E. (2017). Network analysis: A new way of understanding psychopathology? Revista De Psiquiatria Y Salud Mental, 10(4), 206–215. 10.1016/j.rpsm.2017.06.004 28818613

[bibr42-21676968221090039] Fried E. I. Cramer A. O. J. (2017). Moving forward: Challenges and directions for psychopathological network theory and methodology. Perspectives on Psychological Science: A Journal of the Association for Psychological Science, 12(6), 999–1020. 10.1177/1745691617705892 28873325

[bibr43-21676968221090039] Fried E. I. Eidhof M. B. Palic S. Costantini G. Huisman-van Dijk H. M. Bockting C. L. H. Engelhard I. Armour C. Nielsen A. B. S. Karstoft K.-I. (2018). Replicability and generalizability of posttraumatic stress disorder (ptsd) networks: A cross-cultural multisite study of ptsd symptoms in four Trauma patient samples. Clinical Psychological Science, 6(3), 335–351. 10.1177/2167702617745092 29881651 PMC5974702

[bibr44-21676968221090039] Friedman J. Hastie T. Tibshirani R. (2008). Sparse inverse covariance estimation with the graphical lasso. Biostatistics (Oxford, England), 9(3), 432–441. 10.1093/biostatistics/kxm045 18079126 PMC3019769

[bibr45-21676968221090039] Fritz J. De Graaff A. M. Caisley H. Van Harmelen A.-L. Wilkinson P. O. (2018). A systematic review of amenable resilience factors that moderate and/or mediate the relationship between childhood adversity and mental health in young people. Frontiers in Psychiatry, 9, 230. 10.3389/fpsyt.2018.00230. 29971021 PMC6018532

[bibr46-21676968221090039] Fritz J. Fried E. I. Goodyer I. M. Wilkinson P. O. van Harmelen A.-L. (2018). A network model of resilience factors for adolescents with and without exposure to childhood adversity. Scientific Reports, 8(1), 15774. 10.1038/s41598-018-34130-2 30361515 PMC6202387

[bibr47-21676968221090039] Fruchterman T. M. J. Reingold E. M. (1991). Graph drawing by force-directed placement. Journal of Software: Practice and Experience, 21(11), 1129–1164.

[bibr48-21676968221090039] Guyon H. Falissard B. Kop J.-L. (2017). Modeling psychological attributes in psychology – an epistemological discussion: Network analysis versus Latent variables. Frontiers in Psychology, 8, 798. 10.3389/fpsyg.2017.00798. 28572780 PMC5435770

[bibr49-21676968221090039] de Hallen R. V. Jongerling J. Godor B. P. (2020). Coping and resilience in adults: A cross-sectional network analysis. Anxiety, Stress, and Coping, 33(5), 479–496. 10.1080/10615806.2020.1772969 32546008

[bibr50-21676968221090039] Hevey D. (2018). Network analysis: A brief overview and tutorial. Health Psychology and Behavioral Medicine, 6(1), 301–328. 10.1080/21642850.2018.1521283 34040834 PMC8114409

[bibr51-21676968221090039] Höltge J. Theron L. Cowden R. G. Govender K. Maximo S. I. Carranza J. S. Kapoor B. Tomar A. van Rensburg A. Lu S. Hu H. Cavioni V. Agliati A. Grazzani I. Smedema Y. Kaur G. Hurlington K. G. Sanders J. Munford R. Ungar M. (2020). A cross-country network analysis of adolescent resilience. Journal of Adolescent Health, 68(3), 580–588. 10.1016/j.jadohealth.2020.07.010 32919888

[bibr52-21676968221090039] Höltge J. Theron L. van Rensburg A. Cowden R. G. Govender K. Ungar M. (2021). Investigating the interrelations between systems of support in 13- to 18-year-old adolescents: A network analysis of resilience promoting systems in a high and middle-income country. Child Development, 92(2), 586–599. 10.1111/cdev.13483 33480059

[bibr53-21676968221090039] Hoorelbeke K. Marchetti I. De Schryver M. Koster E. H. W. (2016). The interplay between cognitive risk and resilience factors in remitted depression: A network analysis. Journal of Affective Disorders, 195, 96–104. 10.1016/j.jad.2016.02.001 26878206

[bibr54-21676968221090039] Horn J. L. Cattell R. B. (1966). Refinement and test of the theory of fluid and crystallized general intelligences. Journal of Educational Psychology, 57(5), 253–270. 10.1037/h0023816 5918295

[bibr55-21676968221090039] Jefferies P. McGarrigle L. Ungar M. (2018). The CYRM-R: A rasch-validated revision of the child and youth resilience measure. Journal of Evidence-Informed Social Work, 16(1), 1–23. 10.1080/23761407.2018.1548403 30472932

[bibr56-21676968221090039] Jefferies P. Vanstone R. Ungar M. (2021). The rugged resilience measure: Validation of a brief measure of personal resilience. Applied Research in Quality of Life. 10.1007/s11482-021-09953-3.

[bibr57-21676968221090039] Johnson J. S. (2016). Improving online panel data usage in sales research. Journal of Personal Selling & Sales Management, 36(1), 74–85. 10.1080/08853134.2015.1111611

[bibr58-21676968221090039] Jones P. J. (2020). Networktools: Tools for identifying important nodes in networks (1.2.3). [Computer software]. https://CRAN.R-project.org/package=networktools

[bibr59-21676968221090039] Jones P. J. Mair P. McNally R. J. (2018). Visualizing psychological networks: A tutorial in R. Frontiers in Psychology, 9, 1742. 10.3389/fpsyg.2018.01742. 30283387 PMC6156459

[bibr60-21676968221090039] Jones P. J. Ma R. McNally R. J. (2019). Bridge centrality: A network approach to understanding comorbidity. Multivariate Behavioral Research, 56(2), 353–367. 10.1080/00273171.2019.1614898 31179765

[bibr61-21676968221090039] Kalisch R. Cramer A. O. J. Binder H. Fritz J. Leertouwer I. Lunansky G. Meyer B. Timmer J. Veer I. M. van Harmelen A.-L. (2019). Deconstructing and reconstructing resilience: a dynamic network approach. Perspectives on Psychological Science, 14(5), 765–777. 10.1177/1745691619855637. 31365841

[bibr62-21676968221090039] Kan K.-J. de Jonge H. van der Maas H. L. J. Levine S. Z. Epskamp S. (2020). How to compare psychometric factor and network models. Journal of Intelligence, 8(4), 35. 10.3390/jintelligence8040035 33023229 PMC7709577

[bibr63-21676968221090039] Kessler R. C. Walters E. E. (1998). Epidemiology of DSM-III-R major depression and minor depression among adolescents and young adults in the national comorbidity survey. Depression and Anxiety, 7(1), 3–14. 9592628 10.1002/(sici)1520-6394(1998)7:1<3::aid-da2>3.0.co;2-f

[bibr64-21676968221090039] Knutson T. J. Komolsevin R. Chatiketu P. Smith V. R. (2003). A cross-cultural comparison of Thai and US American rhetorical sensitivity: Implications for intercultural communication effectiveness. International Journal of Intercultural Relations, 27(1), 63–78. 10.1016/S0147-1767(02)00060-3

[bibr65-21676968221090039] Kumpfer K. L. (2002). Factors and processes contributing to resilience. In Glantz M. D. Johnson J. L. (Eds.), Resilience and development: positive life adaptations (pp. 179–224). Springer US. 10.1007/0-306-47167-1_9

[bibr66-21676968221090039] Lane J. A. Leibert T. W. Goka-Dubose E. (2017). The impact of life transition on emerging adult attachment, social support, and well-being: A multiple-group comparison. Journal of Counseling & Development, 95(4), 378–388. 10.1002/jcad.12153

[bibr67-21676968221090039] LeBlanc N. J. Brown M. Henin A. (2020). Anxiety disorders in emerging adulthood. In Bui E. Charney M. E. Baker A. W. (Eds.), Clinical handbook of anxiety disorders: From theory to practice (pp. 157–173). Springer International Publishing. 10.1007/978-3-030-30687-8_8

[bibr68-21676968221090039] Liebenberg L. Moore J. C. (2018). A social ecological measure of resilience for adults: The RRC-ARM. Social Indicators Research, 136(1), 1–19. 10.1007/s11205-016-1523-y

[bibr69-21676968221090039] Lunansky G. van Borkulo C. D. Haslbeck J. M. B. van der Linden M. A. Garay C. J. Etchevers M. J. Borsboom D. (2021). The mental health ecosystem: Extending symptom networks with risk and protective factors. Frontiers in Psychiatry, 12, 640658. 10.3389/fpsyt.2021.640658 33815173 PMC8012560

[bibr70-21676968221090039] Madewell A. N. Ponce-Garcia E. (2016). Assessing resilience in emerging adulthood: The resilience scale (RS), connor–davidson resilience scale (CD-RISC), and scale of protective factors (SPF). Personality and Individual Differences, 97, 249–255. 10.1016/j.paid.2016.03.036

[bibr71-21676968221090039] Masten A. S. (2001). Ordinary magic. Resilience processes in development. The American Psychologist, 56(3), 227–238. 10.1037//0003-066x.56.3.227 11315249

[bibr72-21676968221090039] Masten A. S. (2011). Resilience in children threatened by extreme adversity: Frameworks for research, practice, and translational synergy. Development and Psychopathology, 23(2), 493–506. 10.1017/S0954579411000198 23786691

[bibr73-21676968221090039] Masten A. S. Powell J. L. Luthar S. S. (2003). A resilience framework for research, policy, and practice. Resilience and Vulnerability: Adaptation in the Context of Childhood Adversities, 1(25), 153. 10.1017/cbo9780511615788.003

[bibr74-21676968221090039] McNally R. J. (2016). Can network analysis transform psychopathology? Behaviour Research and Therapy, 86, 95–104. 10.1016/j.brat.2016.06.006 27424882

[bibr75-21676968221090039] Mortenson S. T. (2009). Interpersonal trust and social skill in seeking social support among Chinese and Americans. Communication Research, 36(1), 32–53. 10.1177/0093650208326460

[bibr76-21676968221090039] Muthén B. O. (2002). Beyond SEM: General latent variable modeling. Behaviormetrika, 29(1), 81–117.

[bibr77-21676968221090039] Newcomb-Anjo S. E. Barker E. T. Howard A. L. (2017). A person-centered analysis of risk factors that compromise wellbeing in emerging adulthood. Journal of Youth and Adolescence, 46(4), 867–883. 10.1007/s10964-016-0603-2 27826745

[bibr78-21676968221090039] Niffenegger P. Kulviwat S. Engchanil N. (2006). Conflicting cultural imperatives in modern Thailand: Global perspectives. Asia Pacific Business Review, 12(4), 403–420. 10.1080/13602380600571211

[bibr79-21676968221090039] Ong A. D. Bergeman C. S. Boker S. M. (2009). Resilience comes of age: Defining features in later adulthood. Journal of Personality, 77(6), 1777–1804. 10.1111/j.1467-6494.2009.00600.x 19807864 PMC2807734

[bibr80-21676968221090039] Panter-Brick C. (2015). Culture and resilience: Next steps for theory and practice. In Theron L. C. Liebenberg L. Ungar M. (Eds.), Youth resilience and culture: Commonalities and complexities (pp. 233–244). Springer Netherlands. 10.1007/978-94-017-9415-2_17

[bibr81-21676968221090039] R Core Team (2020). R: A language and environment for statistical computing (4.0.0). [Computer software]. R Foundation for Statistical Computing https://www.R-project.org/

[bibr82-21676968221090039] Relman D. A. (2012). The human microbiome: Ecosystem resilience and health. Nutrition Reviews, 70(Suppl 1), S2–S9. 10.1111/j.1753-4887.2012.00489.x 22861804 PMC3422777

[bibr83-21676968221090039] Resilience Research Centre (2003). International resilience project. http://www.resilienceresearch.org/research/projects/international-resilience

[bibr84-21676968221090039] Rhemtulla M. van Bork R. Borsboom D. (2020). Worse than measurement error: Consequences of inappropriate latent variable measurement models. Psychological Methods, 25(1), 30–45. 10.1037/met0000220 31169371

[bibr85-21676968221090039] Robinaugh D. J. Hoekstra R. H. A. Toner E. R. Borsboom D. (2020). The network approach to psychopathology: A review of the literature 2008–2018 and an agenda for future research. Psychological Medicine, 50(3), 353–366. 10.1017/S0033291719003404 31875792 PMC7334828

[bibr86-21676968221090039] Robinaugh D. J. Millner A. J. McNally R. J. (2016). Identifying highly influential nodes in the complicated grief network. Journal of Abnormal Psychology, 125(6), 747–757. 10.1037/abn0000181 27505622 PMC5060093

[bibr87-21676968221090039] Robinson R. (2013). Pathways to resilience in the context of Somali culture and forced displacement—ProQuest. University of Alaska Anchorage. https://search.proquest.com/openview/e08a3945ad0a842848c3f0bb1f332b68/1?pq-origsite=gscholar&cbl=18750&diss=y

[bibr88-21676968221090039] Rook G. A. Lowry C. A. Raison C. L. (2013). Microbial ‘Old Friends’, immunoregulation and stress resilience. Evolution, Medicine, and Public Health, 2013(1), 46–64. 10.1093/emph/eot004 24481186 PMC3868387

[bibr89-21676968221090039] RStudio Team (2020). RStudio: Integrated Development for R (1.2.5042) [Computer software]. RStudio, Inc.

[bibr90-21676968221090039] Rubenstein L. M. Freed R. D. Shapero B. G. Fauber R. L. Alloy L. B. (2016). Cognitive attributions in depression: Bridging the gap between research and clinical practice. Journal of Psychotherapy Integration, 26(2), 103–115. 10.1037/int0000030 27453677 PMC4956086

[bibr91-21676968221090039] Rubin K. H. Cheah C. Menzer M. M. (2010). Peers handbook of cultural developmental science (pp. 223–238). Psychology Press.

[bibr92-21676968221090039] Rutter M. (1985). Resilience in the face of adversity: Protective factors and resistance to psychiatric disorder. The British Journal of Psychiatry, 147(6), 598–611. 10.1192/bjp.147.6.598 3830321

[bibr93-21676968221090039] Schwartz S. J. Petrova M. (2019). Prevention science in emerging adulthood: A field coming of age. Prevention Science, 20(3), 305–309. 10.1007/s11121-019-0975-0 30637671

[bibr94-21676968221090039] Stochl J. Soneson E. Wagner A. P. Khandaker G. M. Goodyer I. Jones P. B. (2019). Identifying key targets for interventions to improve psychological wellbeing: Replicable results from four UK cohorts. Psychological Medicine, 49(14), 2389–2396. 10.1017/S0033291718003288 30430959 PMC6763534

[bibr95-21676968221090039] Theron L. C. Levine D. Ungar M. (2020). African emerging adult resilience: Insights from a sample of township youth. Emerging Adulthood, 9(4), 360–371. 10.1177/2167696820940077

[bibr96-21676968221090039] Theron L. C. Liebenberg L. (2015). Understanding cultural contexts and their relationship to resilience processes. In Theron L. C. Liebenberg L. Ungar M. (Eds.), Youth resilience and culture (pp. 23–36). Springer. 10.1007/978-94-017-9415-2_2

[bibr97-21676968221090039] Thoma M. V. Höltge J. Eising C. M. Pfluger V. Rohner S. L. (2020). Resilience and stress in later life: A network analysis approach depicting complex interactions of resilience resources and stress-related risk factors in older adults. Frontiers in Behavioral Neuroscience, 14, 580969. 10.3389/fnbeh.2020.580969. 33281572 PMC7705246

[bibr98-21676968221090039] Ungar M. (2008). Resilience across cultures. The British Journal of Social Work, 38(2), 218–235. 10.1093/bjsw/bcl343

[bibr99-21676968221090039] Ungar M. (2013). Resilience, Trauma, context, and culture. Trauma, Violence & Abuse, 14(3), 255–266. 10.1177/1524838013487805 23645297

[bibr100-21676968221090039] Ungar M. (2015). Resilience and culture: The diversity of protective processes and positive adaptation. In Theron L. C. Liebenberg L. Ungar M. (Eds.), Youth resilience and culture: Commonalities and complexities (pp. 37–48). Springer Netherlands. 10.1007/978-94-017-9415-2_3

[bibr101-21676968221090039] Ungar M. (2019). Designing resilience research: Using multiple methods to investigate risk exposure, promotive and protective processes, and contextually relevant outcomes for children and youth. Child Abuse & Neglect, 96, 104098. 10.1016/j.chiabu.2019.104098 31376582

[bibr102-21676968221090039] Ungar M. (2021). Multisystemic resilience: Adaptation and transformation in contexts of change. Oxford University Press.

[bibr103-21676968221090039] Ungar M. Brown M. Liebenberg L. Othman R. Kwong W. M. Armstrong M. Gilgun J. (2007). Unique pathways to resilience across cultures. Adolescence, 42(166), 287–310. 17849937

[bibr104-21676968221090039] Ungar M. Theron L. (2020). Resilience and mental health: How multisystemic processes contribute to positive outcomes. The Lancet Psychiatry, 7(5), 441–448. 10.1016/S2215-0366(19)30434-1 31806473

[bibr105-21676968221090039] van Borkulo C. van Bork R. Boschloo L. Kossakowski J. Tio P. Schoevers R. Borsboom D. Waldorp L. (2017). Comparing network structures on three aspects: A permutation test. Preprint Manuscript Submitted for Publication, 1–40. 10.13140/RG.2.2.29455.38569 35404628

[bibr106-21676968221090039] van Bork R. van Borkulo C. D. Waldorp L. J. Cramer A. O. J. Borsboom D. (2018). Network models for clinical psychology. In Stevens’ handbook of experimental psychology and cognitive neuroscience (pp. 1–35). American Cancer Society. 10.1002/9781119170174.epcn518

[bibr107-21676968221090039] van Breda A. D. (2018). A critical review of resilience theory and its relevance for social work. Social Work, 54(1), 1–18. 10.15270/54-1-611

[bibr108-21676968221090039] van den Bosch M. Ode Sang Å. (2017). Urban natural environments as nature-based solutions for improved public health – a systematic review of reviews. Environmental Research, 158, 373–384. 10.1016/j.envres.2017.05.040 28686952

[bibr109-21676968221090039] Vandenbroucke J. P. von Elm E. Altman D. G. Gøtzsche P. C. Mulrow C. D. Pocock S. J. STROBE Initiative (2007). Strengthening the reporting of observational studies in epidemiology (STROBE): explanation and elaboration. Plos Medicine, 4(10), e297. 10.1371/journal.pmed.0040297 17941715 PMC2020496

[bibr110-21676968221090039] Wang D. J. Shi X. McFarland D. A. Leskovec J. (2012). Measurement error in network data: A re-classification. Social Networks, 34(4), 396–409. 10.1016/j.socnet.2012.01.003

[bibr111-21676968221090039] Warren D. E. Dunfee T. W. Li N. (2004). Social exchange in China: The double-edged sword of guanxi. Journal of Business Ethics, 55(4), 353–370. 10.1007/s10551-004-1526-5

[bibr112-21676968221090039] Williams D. R. (2020, March 29). Dealing with negative (red) edges in psychological networks: Frequentist edition, Donald R. W. https://rpubs.com/wdonald1985/591152

[bibr113-21676968221090039] Williams D. R. Rast P. (2020). Back to the basics: Rethinking partial correlation network methodology. British Journal of Mathematical and Statistical Psychology, 73(2), 187–212. 10.1111/bmsp.12173 31206621 PMC8572131

[bibr114-21676968221090039] Williams D. R. Rast P. Pericchi L. R. Mulder J. (2020). Comparing Gaussian graphical models with the posterior predictive distribution and Bayesian model selection. Psychological Methods, 25(5), 653–672. 10.1037/met0000254 32077709 PMC8572134

[bibr115-21676968221090039] Williams D. R. Rhemtulla M. Wysocki A. C. Rast P. (2019). On nonregularized estimation of psychological networks. Multivariate Behavioral Research, 54(5), 719–750. 10.1080/00273171.2019.1575716 30957629 PMC6736701

[bibr116-21676968221090039] Windle G. (2011). What is resilience? A review and concept analysis. Reviews in Clinical Gerontology, 21(2), 152–169. 10.1017/S0959259810000420

[bibr117-21676968221090039] World Health Organization (2013). Consolidated guidelines on the use of antiretroviral drugs for treating and preventing HIV infection. https://www.who.int/publications-detail-redirect/9789241505727 24716260

[bibr118-21676968221090039] Wu Q. Tsang B. Ming H. (2014). Social capital, family support, resilience and educational outcomes of Chinese migrant children. The British Journal of Social Work, 44(3), 636–656. 10.1093/bjsw/bcs139

[bibr119-21676968221090039] Zhang J. Zhang J. Zhou M. Yu N. X. (2018). Neighborhood characteristics and older adults’ well-being: The roles of sense of community and personal resilience. Social Indicators Research, 137(3), 1–15. 10.1007/s11205-017-1626-0

