# Supplementary material for: A Cross-Country Network Analysis of Resilience Systems in Young Adults
Source: Emerg Adulthood. 2022 May 2;11(2):415–30. doi: 10.1177/21676968221090039 (PMC10009297; doi:10.1177/21676968221090039)
Supplement: sj-pdf-1-eax-10.1177_21676968221090039 – Supplemental Material for A Cross-Country Network Analysis of Resilience Systems in Young Adults [file sj-pdf-1-eax-10.1177_21676968221090039.pdf]

Supplemental Information

List of packages used in data preparation and analyses:

- BGGM v2.0.3
- dplyr v1.0.0
- expss v0.10.2
- ggplot2 v3.3.0
- Matrix v1.2-18
- matrixStats v0.58.0
- networktools v1.2.3
- psych v1.9.12.31
- psychonetrics v0.8.1
- qgraph v1.6.5
- readxl v1.3.1
- reshape2 v1.4.4
- tidyverse v1.3.0
- NetworkComparisonTest (GitHub commit 14.10.2020; downloaded 02.03.2021; minor adaptations made by the lead author to involve pruned models and to test for global bridge expected influence; script available upon request)

Table S1. Fit indices for pruned models (.05, recursive)

**Network**	**DF**	**AIC**	**BIC**	**RMSEA**	**CFI**	**TLI**	**ChiSq**
Brazil	278	46642.79	47211.56	.039	.96	.95	553.22
China	277	49508.56	50092.55	.045	.93	.92	97.34
Indonesia	273	38822.59	39397.69	.032	.98	.97	436.63
Russia	276	46600.35	47182.37	.039	.96	.95	555.33
Thailand	276	42061.60	42629.45	.043	.95	.94	582.58
US	279	45740.78	46298.72	.050	.92	.90	711.23
Vietnam	279	46734.59	47298.50	.040	.95	.94	564.50

Note: DF=degrees of freedom; AIC=Akaike information criterion; BIC=Bayesian information criterion; RMSEA=Root mean square error of approximation; CFI=Comparative fit index; TLI=Tucker-Lewis index; ChiSq=Chi-square statistic.

Table S2. Edge weights for the pruned networks.

(1) Brazil

	**R1**	**R2**	**R3**	**R4**	**R5**	**R6**	**R7**	**R8**	**R9**	**R10**	**A1**	**A2**	**A3**	**A4**	**A5**	**A6**	**A7**	**A8**	**A9**	**A10**	**A11**	**A12**	**A13**	**A14**	**A15**	**A16**	**A17**
**R1**	-	0	.105	.154	0	.124	0	.095	.153	.226	0	0	0	0	.114	0	.183	0	0	0	0	-.078	0	0	0	0	0
**R2**		-	.215	0	.277	.16	0	0	.17	0	0	0	0	0	0	0	0	0	0	0	0	0	0	0	0	0	0
**R3**			-	.234	0	0	.173	0	.179	0	0	.147	0	0	0	0	0	0	0	0	0	0	0	0	0	0	0
**R4**				-	0	.28	0	0	0	0	.153	0	0	0	0	0	0	0	0	0	.108	0	0	0	0	0	0
**R5**					-	0	.183	0	.159	.227	0	0	.172	0	0	0	0	0	0	0	0	0	0	.143	0	0	0
**R6**						-	.121	.119	0	.246	0	0	0	0	0	0	0	0	0	0	0	0	0	0	0	0	0
**R7**							-	0	.126	0	0	0	0	0	0	0	0	0	0	0	0	0	0	0	0	0	0
**R8**								-	0	.163	0	.162	0	0	0	.114	0	0	0	0	0	0	0	.149	.124	0	0
**R9**									-	0	0	0	0	0	0	0	0	0	0	0	.095	0	0	0	0	0	0
**R10**										-	0	0	0	0	0	0	0	0	0	0	0	0	0	0	0	0	0
**A1**											-	.195	.193	0	0	0	0	0	0	0	0	0	.157	.202	0	0	0
**A2**												-	.155	0	0	.214	0	0	0	0	0	0	0	0	0	.166	0
**A3**													-	0	0	0	.242	0	0	0	0	0	0	0	0	0	0
**A4**														-	.197	0	0	0	0	.147	.333	0	0	.165	.139	0	0
**A5**															-	0	0	.251	0	.249	0	0	0	0	0	0	.195
**A6**																-	0	0	0	0	.131	0	0	0	.132	.124	0
**A7**																	-	.146	.118	0	0	.194	0	0	.134	0	0
**A8**																		-	.096	0	0	0	0	0	.189	0	0
**A9**																			-	.147	0	.482	.121	.083	0	0	0
**A10**																				-	0	0	.288	0	0	0	.116
**A11**																					-	0	0	0	.18	0	.189
**A12**																						-	0	0	0	.131	0
**A13**																							-	0	.089	.14	0
**A14**																								-	0	.241	0
**A15**																									-	0	.234
**A16**																										-	0
**A17**																											-

Note: Rx=RRM item; Ax=ARM item.

(2) China

	R1	R2	R3	R4	R5	R6	R7	R8	R9	R10	A1	A2	A3	A4	A5	A6	A7	A8	A9	A10	A11	A12	A13	A14	A15	A16	A17
**R1**	-	.144	0	.165	0	.12	0	.158	.194	0	0	0	0	0	0	0	0	0	0	0	0	0	0	0	0	0	0
**R2**		-	.147	.104	.178	.11	0	0	0	.135	.227	0	0	0	0	0	0	0	0	0	0	0	0	0	0	0	0
**R3**			-	.153	0	0	.151	0	.267	0	0	0	.157	0	0	0	0	0	0	0	0	0	0	0	0	0	0
**R4**				-	0	.159	.158	0	0	0	0	0	0	0	0	0	0	0	0	0	.19	0	0	0	0	0	0
**R5**					-	.162	0	.141	.167	.155	0	0	0	0	0	0	0	0	0	0	0	0	0	0	0	0	0
**R6**						-	0	.133	0	0	0	0	0	0	0	0	0	0	0	0	0	0	0	0	0	.191	0
**R7**							-	0	0	.164	0	0	0	0	0	0	0	0	.123	0	0	0	.176	0	0	0	0
**R8**								-	.11	0	0	0	.159	0	0	0	0	0	0	0	0	0	0	0	0	0	0
**R9**									-	.164	0	0	0	0	0	0	0	0	.161	0	0	0	0	0	0	0	0
**R10**										-	0	0	0	0	0	0	0	0	0	0	.176	0	0	0	0	0	0
**A1**											-	.231	0	0	0	0	.237	0	0	.151	0	0	0	0	0	0	.142
**A2**												-	0	0	0	.14	0	.158	.163	0	.13	0	0	0	0	0	0
**A3**													-	0	0	0	.153	0	0	.133	0	.111	0	0	0	.247	0
**A4**														-	0	.192	0	0	.163	0	0	.154	.142	.245	.157	0	.118
**A5**															-	0	0	.26	.169	.144	0	.141	0	0	0	.196	.102
**A6**																-	0	.164	0	0	.156	0	0	0	.113	0	0
**A7**																	-	0	0	.142	0	.209	0	0	0	0	0
**A8**																		-	0	0	0	0	0	0	.165	0	0
**A9**																			-	0	0	0	0	0	.156	0	0
**A10**																				-	.131	0	0	0	0	0	.132
**A11**																					-	.159	.169	0	.157	0	0
**A12**																						-	0	0	0	0	.142
**A13**																							-	0	0	.207	.141
**A14**																								-	0	.298	0
**A15**																									-	0	.169
**A16**																										-	0
**A17**																											-

Note: Rx=RRM item; Ax=ARM item.

(3) Indonesia

	R1	R2	R3	R4	R5	R6	R7	R8	R9	R10	A1	A2	A3	A4	A5	A6	A7	A8	A9	A10	A11	A12	A13	A14	A15	A16	A17
**R1**	-	.129	0	0	0	.165	0	0	.218	.213	0	0	0	0	0	0	0	0	0	0	.11	0	0	0	.089	0	0
**R2**		-	.13	.206	.144	0	0	0	0	.13	0	.147	.148	0	0	0	.1	0	0	0	-.145	0	0	0	0	0	0
**R3**			-	0	.184	0	.248	0	.261	.2	0	0	0	0	0	0	0	0	0	0	0	0	0	0	0	0	0
**R4**				-	0	.251	0	0	.212	0	0	0	0	0	0	.136	0	.113	0	0	0	0	0	0	0	0	0
**R5**					-	.168	0	0	0	0	0	0	.1	0	0	0	.116	0	0	0	0	0	0	0	0	.134	0
**R6**						-	0	0	0	.159	0	0	0	0	0	0	0	0	0	0	0	0	0	.158	0	0	0
**R7**							-	0	0	0	0	0	.156	.131	0	0	0	0	0	0	0	0	0	0	0	0	0
**R8**								-	.205	.203	0	.152	0	0	.126	0	0	0	0	0	.128	0	0	0	0	0	0
**R9**									-	0	0	0	0	0	0	0	0	0	0	.101	0	0	0	0	0	.156	0
**R10**										-	0	0	0	0	0	0	0	0	0	0	0	0	0	0	0	0	.169
**A1**											-	0	0	0	.126	.125	0	.156	0	.185	0	.119	.218	0	0	0	0
**A2**												-	0	0	0	.184	0	0	0	.105	0	0	0	0	0	.147	0
**A3**													-	0	0	0	0	0	0	.095	.117	0	.172	.151	0	.108	0
**A4**														-	.178	0	0	0	.109	0	.156	0	0	0	.238	0	.241
**A5**															-	0	0	.19	0	0	.156	0	0	0	0	0	.162
**A6**																-	0	0	0	0	.119	0	0	.129	0	0	0
**A7**																	-	0	.213	.203	0	0	.194	0	0	0	0
**A8**																		-	0	0	.164	0	0	0	.184	0	0
**A9**																			-	0	.137	.387	.2	0	0	0	0
**A10**																				-	0	.122	.171	.192	0	0	0
**A11**																					-	0	0	0	.227	0	0
**A12**																						-	0	0	0	.141	.163
**A13**																							-	0	0	0	0
**A14**																								-	0	.193	0
**A15**																									-	.181	0
**A16**																										-	0
**A17**																											-

Note: Rx=RRM item; Ax=ARM item.

(4) Russia

	R1	R2	R3	R4	R5	R6	R7	R8	R9	R10	A1	A2	A3	A4	A5	A6	A7	A8	A9	A10	A11	A12	A13	A14	A15	A16	A17
**R1**	-	0	0	0	0	.26	0	.213	.168	.185	0	0	.116	0	0	0	0	0	0	0	0	0	0	0	0	0	.127
**R2**		-	.133	0	.321	.177	0	0	0	0	.129	0	.176	0	0	0	0	0	0	0	0	0	0	0	0	0	0
**R3**			-	0	.212	.186	0	0	.145	0	.09	0	0	0	0	0	0	0	0	0	0	0	0	.14	0	.099	0
**R4**				-	0	.207	0	.122	.26	.131	0	.137	0	0	0	0	.123	0	0	0	0	0	0	.158	0	0	0
**R5**					-	0	.158	0	.199	0	0	0	0	0	0	0	0	0	0	0	0	0	0	0	0	0	0
**R6**						-	0	0	0	.204	0	0	0	0	0	0	0	0	0	0	0	0	0	0	0	0	0
**R7**							-	0	.214	0	0	0	0	0	0	0	0	0	0	0	0	0	0	0	0	0	0
**R8**								-	0	.147	0	0	0	0	0	0	0	0	0	0	0	0	0	0	0	.174	0
**R9**									-	0	0	0	0	0	0	0	0	0	0	0	0	0	0	0	-.142	0	.106
**R10**										-	0	0	0	0	0	0	.113	.115	0	.109	0	0	0	0	0	0	0
**A1**											-	.163	0	.129	0	0	0	0	0	.128	0	.147	.122	0	0	.113	0
**A2**												-	0	0	0	.127	0	0	0	0	0	0	0	.128	0	.1	0
**A3**													-	0	0	.174	0	0	0	.214	0	0	0	0	0	.147	0
**A4**														-	.135	0	0	.167	.367	0	.189	0	0	0	0	0	0
**A5**															-	0	0	.248	.153	0	.133	0	0	0	0	.125	.142
**A6**																-	0	0	0	0	0	0	.134	.171	0	0	0
**A7**																	-	0	0	.165	.107	0	.245	0	0	0	0
**A8**																		-	0	0	0	.149	0	0	0	0	.184
**A9**																			-	0	.21	0	0	0	.166	0	.147
**A10**																				-	0	.189	.232	0	0	0	0
**A11**																					-	.168	0	0	.242	0	0
**A12**																						-	0	0	0	0	0
**A13**																							-	0	.116	.191	0
**A14**																								-	.134	.205	0
**A15**																									-	0	.242
**A16**																										-	0
**A17**																											-

Note: Rx=RRM item; Ax=ARM item.

(5) Thailand

	R1	R2	R3	R4	R5	R6	R7	R8	R9	R10	A1	A2	A3	A4	A5	A6	A7	A8	A9	A10	A11	A12	A13	A14	A15	A16	A17
**R1**	-	.173	0	0	.158	.194	0	0	0	.109	0	0	0	0	0	0	0	0	0	0	0	0	0	0	0	.183	0
**R2**		-	0	.263	0	0	.184	.182	0	.133	0	0	0	0	0	0	.122	0	0	0	0	0	0	0	0	0	0
**R3**			-	.14	.117	0	0	.135	0	.148	0	0	0	0	0	0	0	0	0	0	0	0	0	.139	0	.123	0
**R4**				-	.116	0	0	0	.21	0	0	0	0	0	0	0	0	0	0	0	0	0	0	0	0	.147	0
**R5**					-	0	.183	0	0	0	0	0	.183	0	0	0	0	0	0	0	0	0	.11	0	0	0	.108
**R6**						-	0	.296	.211	.127	0	0	0	0	0	0	0	0	0	0	0	0	0	0	0	0	0
**R7**							-	0	.195	0	0	0	0	0	0	0	0	0	0	0	0	0	0	0	0	0	0
**R8**								-	0	0	0	.146	0	0	.168	.205	-.139	0	0	0	0	0	0	0	0	0	0
**R9**									-	.167	0	0	0	0	0	0	0	0	0	0	0	0	0	0	0	0	0
**R10**										-	.121	0	0	0	0	0	.119	.124	0	0	0	0	0	0	0	0	0
**A1**											-	0	.142	0	0	.162	0	.145	0	.229	0	.136	0	0	0	0	0
**A2**												-	.184	0	0	0	0	0	0	0	0	.133	0	.211	.138	0	.195
**A3**													-	0	0	.118	0	0	.146	0	0	0	0	0	0	.19	0
**A4**														-	.315	0	0	.202	0	0	.276	0	0	0	.14	0	0
**A5**															-	0	0	0	0	0	.148	0	0	.147	.161	0	0
**A6**																-	0	0	0	0	0	0	0	.16	.193	0	0
**A7**																	-	0	.135	.179	0	0	.136	.244	0	.126	.119
**A8**																		-	.162	0	.175	0	0	0	0	0	.227
**A9**																			-	.142	0	.34	0	0	0	0	0
**A10**																				-	0	0	.258	0	0	.145	0
**A11**																					-	0	0	.169	.18	0	0
**A12**																						-	.165	0	0	0	0
**A13**																							-	0	0	0	.163
**A14**																								-	0	0	0
**A15**																									-	.144	0
**A16**																										-	0
**A17**																											-

Note: Rx=RRM item; Ax=ARM item.

(6) US

	R1	R2	R3	R4	R5	R6	R7	R8	R9	R10	A1	A2	A3	A4	A5	A6	A7	A8	A9	A10	A11	A12	A13	A14	A15	A16	A17
**R1**	-	0	.134	0	0	.221	.14	0	0	.134	0	0	0	.121	0	0	.161	0	0	0	0	0	0	0	0	.167	0
**R2**		-	.171	.191	.265	0	.115	0	.16	0	.115	0	0	0	0	0	0	0	0	0	0	0	0	0	0	0	0
**R3**			-	0	.152	0	.119	0	.152	.135	0	0	.141	0	0	0	0	0	0	0	0	0	0	0	0	0	0
**R4**				-	0	.149	0	0	.188	.15	0	0	0	0	0	0	0	0	0	0	.139	0	0	0	0	0	0
**R5**					-	.106	0	.193	0	.118	0	0	0	0	0	0	0	0	0	0	0	0	0	0	0	0	0
**R6**						-	0	.185	.143	.151	0	0	0	0	0	0	0	0	0	0	0	0	0	.133	0	0	0
**R7**							-	0	0	0	0	0	0	0	0	0	0	0	0	.107	0	.085	.127	0	.093	0	0
**R8**								-	0	0	0	0	0	0	0	.208	0	0	0	0	0	0	0	0	0	0	0
**R9**									-	0	0	.143	0	0	0	0	0	0	0	0	0	0	0	0	0	0	0
**R10**										-	0	0	0	0	0	.082	0	.176	0	0	0	0	0	.162	0	0	0
**A1**											-	.119	.168	.191	0	0	0	0	0	0	0	.136	0	.187	0	.103	0
**A2**												-	0	0	0	.16	0	0	0	0	0	.128	0	.174	0	.191	0
**A3**													-	0	0	0	.12	0	0	0	0	0	.167	.17	0	.13	0
**A4**														-	.299	0	0	0	0	0	.197	0	0	0	0	0	.17
**A5**															-	0	.215	0	0	0	0	0	0	0	.221	0	0
**A6**																-	0	0	0	0	0	0	0	0	.191	0	.18
**A7**																	-	0	0	.233	0	.219	.151	0	0	0	0
**A8**																		-	0	.173	.115	0	0	0	.148	0	.181
**A9**																			-	0	0	.501	0	0	0	0	0
**A10**																				-	0	0	.212	0	0	.178	0
**A11**																					-	0	0	0	.394	0	.16
**A12**																						-	0	0	0	0	0
**A13**																							-	0	0	0	0
**A14**																								-	0	0	0
**A15**																									-	0	0
**A16**																										-	.17
**A17**																											-

Note: Rx=RRM item; Ax=ARM item.

(7) Vietnam

	R1	R2	R3	R4	R5	R6	R7	R8	R9	R10	A1	A2	A3	A4	A5	A6	A7	A8	A9	A10	A11	A12	A13	A14	A15	A16	A17
**R1**	-	0	0	.165	0	.186	0	.221	0	.19	0	0	.122	0	0	0	0	0	0	0	0	0	0	0	0	0	0
**R2**		-	.174	.135	0	0	0	0	.19	.14	0	0	0	0	0	0	0	0	0	0	0	0	0	.235	0	0	0
**R3**			-	0	.17	0	.178	.204	.196	0	0	0	0	0	0	0	0	0	0	0	0	0	0	0	0	.189	0
**R4**				-	0	.136	0	0	.221	0	.148	0	0	0	0	0	0	0	0	0	0	0	0	0	0	0	0
**R5**					-	.099	0	.119	.174	.14	0	0	.165	0	0	0	0	0	0	0	0	0	0	0	0	0	0
**R6**						-	0	.1	.115	.104	0	0	0	0	0	.145	0	0	0	0	.102	0	0	0	0	0	0
**R7**							-	0	0	0	0	0	.144	0	.121	0	0	0	0	0	0	0	0	0	0	0	0
**R8**								-	0	0	0	0	0	0	0	0	0	.125	0	0	0	0	0	0	0	0	0
**R9**									-	.126	0	0	0	0	0	0	0	0	0	0	0	0	0	0	0	0	0
**R10**										-	0	0	0	.111	0	0	0	0	0	0	0	0	.14	0	0	0	0
**A1**											-	.198	.178	0	0	0	.126	0	.183	.127	0	0	0	0	0	0	0
**A2**												-	0	0	0	0	0	0	0	0	0	0	0	0	0	0	.202
**A3**													-	0	0	0	.155	0	0	0	0	0	.119	.256	0	.256	0
**A4**														-	.217	0	0	0	0	0	.266	0	0	0	.191	0	.162
**A5**															-	0	0	.182	0	0	.183	.13	0	0	0	0	.161
**A6**																-	0	0	0	0	.228	0	0	.216	0	0	0
**A7**																	-	.155	0	.144	0	.143	.114	0	0	0	0
**A8**																		-	0	.087	0	0	0	0	.123	.205	.146
**A9**																			-	.196	0	.381	0	0	0	0	0
**A10**																				-	.139	0	.155	0	0	0	.087
**A11**																					-	0	0	0	.222	0	0
**A12**																						-	.211	0	.127	0	0
**A13**																							-	0	0	0	.132
**A14**																								-	0	0	0
**A15**																									-	0	.163
**A16**																										-	0
**A17**																											-

Note: Rx=RRM item; Ax=ARM item.

Table S3. Percentage of sample model edges appearing in 25% case-drop bootstrapped models (n=1,000).

(1) Brazil

	**1.**	**2.**	**3.**	**4.**	**5.**	**6.**	**7.**	**8.**	**9.**	**10.**	**11.**	**12.**	**13.**	**14.**	**15.**	**16.**	**17.**	**18.**	**19.**	**20.**	**21.**	**22.**	**23.**	**24.**	**25.**	**26.**	**27.**
**R1**	-	9.8	52.5	85.1	2.2	94.9	2.5	61.5	59.5	99.6	0.5	0	0.1	0.1	49.5	0	68.2	0.7	15.3	3.2	0	55.6	0	0	1.1	0.9	0
**R2**		-	93.2	14.6	100	58.7	2.2	0.4	86.1	16.7	0.2	1	8	6.6	0	0	2.6	7.4	1.5	0	0.2	0.3	0	1.7	0	28.7	0.7
**R3**			-	99.4	21.8	0	93.7	1.6	99.1	0.6	24.8	50.4	14.4	0.1	0	0	0.6	0	1.9	0	0	0.9	0	0.2	3.8	0.1	0
**R4**				-	27.5	100	3.3	3.5	2.7	3.7	42.9	18.4	0.1	0	0	0.6	0.2	0	0	0.1	57.2	0	0	6.4	1.5	13.1	0
**R5**					-	0	98.7	0	83	99	2.6	0	61.5	0	0	0	0	0.1	0	0.4	0	0	9	39.3	6	7.5	4.2
**R6**						-	76.1	45.8	0.3	99.9	0	0	16.7	0	0	21.2	0	0	0	0.2	1.5	0	1.4	0	1.5	0	15.1
**R7**							-	0	69.2	0	0	0	2.7	0	0.1	0	0.1	7.2	0.7	1.3	0	3.1	17.3	0	0	0.1	0
**R8**								-	29.1	90.9	0.4	96.7	0	0	9.5	53.5	0.1	2.9	0	2.2	0	5.9	0	86.1	65.7	0.5	0
**R9**									-	26.4	0	20.1	0.5	0.9	0	0.5	16.5	0.2	0	22.8	40.1	0	14.6	0.1	0.3	0.4	3.7
**R10**										-	0	0	0	0	0.3	0.1	18.1	0	13.5	0	1	23.9	2.2	0	0	0.5	0
**A1**											-	99.5	84.2	0	0	0.6	34.6	18	0	0.2	11.5	5.9	94.1	98.6	6.3	0	3.9
**A2**												-	81.7	1.2	0	99.9	0	0	0	0	14	0	0	0	1.3	71.8	18.3
**A3**													-	2.8	12.4	20.9	99.7	0.3	0.6	0.9	13.1	0	0.4	0.1	1.8	5.2	1.2
**A4**														-	93.6	0	3.2	0.3	0.1	91.2	100	0.9	20.7	43.1	88.6	0	23.3
**A5**															-	0	0	100	0.1	100	40	0	0	0.8	5.5	18.8	98.3
**A6**																-	0	0	0	0	54.8	24.7	0	37	92.5	27.6	1
**A7**																	-	71.8	48.2	0	0	99.3	8.9	0.2	37.4	22.9	3.1
**A8**																		-	36.3	3.6	6.6	0	0	0	99.6	1.7	1.2
**A9**																			-	58.4	23.7	100	86.1	40.8	2	0	0.1
**A10**																				-	0	28.4	100	3.1	0	0.7	48.6
**A11**																					-	1.7	0.1	17.6	99.1	2.8	66.8
**A12**																						-	0	0	0	38	0.2
**A13**																							-	0	30.3	61.2	2.9
**A14**																								-	2	97.2	18.3
**A15**																									-	0.3	99.9
**A16**																										-	0.8
**A17**																											-

Note: Rx=RRM item; Ax=ARM item.

Average of 76.04% of edges present in sample model also present in boots; 5.61% of edges not present in sample model but present in boots.

(2) China

	1.	2.	3.	4.	5.	6.	7.	8.	9.	10.	11.	12.	13.	14.	15.	16.	17.	18.	19.	20.	21.	22.	23.	24.	25.	26.	27.
**R1**	-	71.3	0.1	88	1.7	63	0.7	84.1	98	25.7	5.8	0.1	5.5	0.4	0.2	0	13.6	2.8	0	1.2	0	0.1	1.3	2.6	0.3	0.2	0
**R2**		-	76.4	36.7	97.9	56.3	32.7	0.2	33.8	44.8	97.7	11.5	0	0	0.1	0.1	2.9	0.6	0.1	0.1	0.6	0.1	0.6	0.7	0	9.8	0
**R3**			-	88.3	2.8	5.2	38.4	30.2	100	0	1.7	0	83.7	0.6	1.6	21.3	0	0.1	0.9	0.1	0	20	0	0.1	17.1	0	0.1
**R4**				-	15.4	75.4	60.4	1.2	2.4	4.6	9.4	0	0	11.4	0	0	10.4	0.2	0.1	0.1	99.5	0.3	0	2.3	2.5	0.2	0.4
**R5**					-	80.5	8.9	59.7	91.3	53.2	0	1.5	23.7	0.3	0	0	0	0.2	0	13.7	0.2	0	0.8	25.9	0.3	0	4.2
**R6**						-	20.1	81.7	1.9	4.8	0.2	1.3	0	2.1	0	0	0	0	0	0	3.8	0.4	0	0.7	28.6	87.7	0
**R7**							-	24.1	10.8	32.9	2.3	0	5	1.7	0	8	0.7	0	33.9	0.1	11.7	0.3	93.2	1.1	0	0.9	0
**R8**								-	30.1	1.5	5.9	4.9	53.9	0	18.5	5.6	0.2	4.8	0	0	0.3	11.2	1.2	1.4	0	0.3	5.5
**R9**									-	84.9	0.1	11.2	3.6	3	0.1	1.4	7.3	0.2	65.3	0.2	0.1	0.1	0.1	0.2	3.5	8.7	0.7
**R10**										-	0.7	6.4	0	0.2	0.1	26.6	18.2	0.9	0	1.2	39.9	0	1.3	1.1	0.2	1	0.1
**A1**											-	85.5	15.6	0.1	0	2.8	70.2	4.3	0.2	87.9	0.1	18.7	0.2	7.9	9.8	10.6	39.2
**A2**												-	2.2	0.3	0	75.7	0	63.7	60.6	0.9	37.9	25.8	21.9	23.7	0.1	30.5	0.4
**A3**													-	0	0	0.2	35.5	23.8	0.2	62.4	5.1	48.4	0.1	15.8	2.2	100	0
**A4**														-	11	94.4	0.1	6.5	58.6	24	9	70.3	50	37.6	91	2.7	76.2
**A5**															-	4.9	5.3	100	65.3	67.9	6.5	71.9	0.3	0.1	0.4	94.3	46.7
**A6**																-	10	53.6	20.9	1.2	70.2	0.1	0	0.5	47.3	20.5	2.4
**A7**																	-	0.1	19.2	45.3	0.5	81.7	17.5	28.7	9.7	1.8	17.3
**A8**																		-	31.4	0.7	0.2	0.7	9.1	6.9	69.9	0.1	1.3
**A9**																			-	0.7	0.1	23.4	0	12.4	66.4	16.5	3.6
**A10**																				-	41.3	1.9	2	7.1	0	12.4	72.9
**A11**																					-	82	77.5	15.4	82.9	2.6	6.1
**A12**																						-	16.9	0.1	1.4	0.1	85.2
**A13**																							-	4.5	2.7	71.8	67.9
**A14**																								-	6.9	91.8	0.9
**A15**																									-	0.5	96.6
**A16**																										-	13.2
**A17**																											-

Note: Rx=RRM item; Ax=ARM item.

Average of 69.51% of edges present in model also present in boots; 8.27% of edges not present in model but present in boots.

(3) Indonesia

	1.	2.	3.	4.	5.	6.	7.	8.	9.	10.	11.	12.	13.	14.	15.	16.	17.	18.	19.	20.	21.	22.	23.	24.	25.	26.	27.
**R1**	-	48.5	2.5	0.2	4.3	94.4	32.6	29.9	99.3	99.7	0	0	0	22.9	0.1	2.4	0	2.5	0	0	31	0.8	0.5	0	27.9	0	0.3
**R2**		-	67.1	99	74.4	0	0.3	8.8	22.8	53.9	1.1	53.3	72.5	0.1	0	0.9	51	9.4	0	8.5	30.3	0.3	0.8	23.1	2.7	0	4
**R3**			-	0	39.7	56.8	98.8	0	99.9	92.5	8	2.4	0.5	0.3	0	1.6	0.4	2.9	0	0.1	0	0.1	1	0.5	7.1	7.3	0.9
**R4**				-	3.3	100	8	2.7	98.2	26.7	0.5	37.8	0.1	0.2	0	32	7.9	48.4	0	4.6	0	0	4.5	0	16.7	0.7	0.9
**R5**					-	76.5	13.2	9.8	33.3	5.9	11.8	0	54.4	0	0.1	0.2	62.4	12.9	0	0	3.7	0	0	0.2	0	29.1	1.1
**R6**						-	0	0.9	0.2	46.3	0.1	0	4	0	0.4	0.4	0.1	0.3	1.5	0.2	13.2	0.1	0.5	75.5	15.6	51.6	0
**R7**							-	7.9	4.4	4.3	0.2	0	35.3	64	0	0.1	16.5	0.8	0.1	0	0.6	0	18.4	0.2	0.1	0	0
**R8**								-	75.5	77	0	52.4	4.5	26.2	36.8	0.1	0.7	6.6	0	0.4	64	0	0.1	2.6	0.2	33	0.1
**R9**									-	0.2	2.9	1.6	0.3	4.2	1.7	0.3	3.6	0	0.8	82.5	0	0	0.3	19.4	1.2	61.3	0.4
**R10**										-	0	0.1	2.5	5.4	0.1	3.9	0	12	0	0	0.1	0.1	0	19.6	0	0.9	87.4
**A1**											-	0	1.7	2.5	76	91.2	18.4	63.4	0	95.3	0.7	46.4	98.8	3.1	0.1	2.3	3.5
**A2**												-	12.6	29.3	0	97.6	0	0	3.8	34.7	0.3	0.5	0.6	1.7	6.3	35.9	33.7
**A3**													-	0	0	0.4	10.8	0.1	2.7	69.4	61.3	0.6	84.8	52.5	22.4	46.7	1.1
**A4**														-	97.9	0	0.3	0	58.2	0	81.3	0.2	2.2	0.2	100	0.1	99.8
**A5**															-	0.1	10.1	99.5	3.5	0.2	89.8	3	1.1	0.8	4.1	0	75.2
**A6**																-	0	2.8	0	1.2	88.5	0	0	63.6	2.9	5.2	0.4
**A7**																	-	0.5	98.3	96.1	0.1	23.2	93.1	23.8	0	2.4	0
**A8**																		-	0	26	95.2	0	9.7	6.1	97	0.2	0
**A9**																			-	0.3	68.4	100	99.8	0	0.9	25.4	4
**A10**																				-	0	80.1	92.1	73.1	0.1	0	1.1
**A11**																					-	36.5	0.3	0.1	100	0	20.3
**A12**																						-	0.2	0.7	2.4	57.8	44.5
**A13**																							-	22.8	10.8	0	2.4
**A14**																								-	0.1	87.1	0.1
**A15**																									-	94.7	6.7
**A16**																										-	0.2
**A17**																											-

Note: Rx=RRM item; Ax=ARM item.

Average of 72.77% of edges present in model also present in boots; 5.98% of edges not present in model but present in boots.

(4) Russia

	1.	2.	3.	4.	5.	6.	7.	8.	9.	10.	11.	12.	13.	14.	15.	16.	17.	18.	19.	20.	21.	22.	23.	24.	25.	26.	27.
**R1**	-	8.4	8.1	17.7	21.7	100	0	99.3	50.3	99.2	0.3	0	30	0	0	0	0.7	0.2	1.2	6.6	17.6	0.1	0.1	0	0.7	34.2	35.9
**R2**		-	83.2	31.3	100	91.4	11.9	0	19.5	0.5	52.9	1.9	72.3	0	0	10.4	13.5	8.1	0	5.3	4.4	1.2	0.1	23.6	6.3	0	3.4
**R3**			-	18.5	99.2	98.7	22.1	0	77.6	0.1	30.2	0	4.7	0.3	6.3	0	0	0	1.5	0.3	0.6	0	0	39.3	7	62.6	0.2
**R4**				-	11.6	94.8	8.5	66.7	99.6	91.6	0.2	67	4.6	0.1	0	21.3	67.5	4.3	11.3	0.1	0.9	0.2	0	52.6	0	0	0.1
**R5**					-	1.1	42.7	5.2	78.2	1.5	0.2	2.9	6.9	12.6	0.1	0.1	0	0	0	0	0	0	9.1	5	0	4.7	0
**R6**						-	0	8	31.3	100	1.5	23.4	4.4	0.2	10.1	1.7	2.7	1.4	0	0	0	0	10.8	0	14.6	0	0
**R7**							-	0.6	98.7	0	19	2.5	19.5	0	1	0.2	0	8.2	0	11.8	0.5	0	0.3	0	0.1	1.4	0.1
**R8**								-	0.3	86.7	0	0.1	0	0.3	0.2	0	1	2.6	0	0	0.1	11.6	0.7	0.4	0.1	91.7	6.6
**R9**									-	0	0.4	13.2	29.6	0.2	0.2	0.7	1.1	2.4	0.2	1.8	2.3	8.8	0.2	1.2	93	0	81.2
**R10**										-	0	0.3	0.3	0	0.2	0	80.1	35	5	39.7	0	2.8	0.4	13	0.1	13	0.5
**A1**											-	95.8	16.8	89.8	0.3	12.9	12.9	0.1	0	60.3	0.3	91.3	38	21.1	0.4	54.2	6.9
**A2**												-	0.1	0	16.1	68	0	0	0	0.2	0	5.9	1.1	81.3	6	58.8	0.6
**A3**													-	0.2	14	98	5	0.1	0.1	93.1	0	7.7	8.6	0	0.1	34.5	0.6
**A4**														-	79.5	3.6	5.1	87	100	0	97.5	1.7	0.3	0	25.9	0.2	7.2
**A5**															-	0	1.3	100	95.7	10.2	53.6	0.2	0.3	0.3	41.5	68.2	66.3
**A6**																-	0.7	0.3	6.8	2.8	0.4	7.1	64.6	74.1	10.2	19	5.6
**A7**																	-	3	0	79.1	62.7	13.8	100	14.5	3.4	0	0.1
**A8**																		-	1.3	33.9	0.5	67.1	0.1	14.6	33.6	31.3	90.8
**A9**																			-	0	99.9	1.2	0	0.5	74.5	0	49.1
**A10**																				-	0.5	92.8	100	4.9	4	0	21.7
**A11**																					-	94.6	1	0.1	99.8	0	48.1
**A12**																						-	0.2	1.7	0.4	0	0
**A13**																							-	23.6	61	99.3	22.2
**A14**																								-	41.5	93.6	0.3
**A15**																									-	0.8	99.6
**A16**																										-	5.2
**A17**																											-

Note: Rx=RRM item; Ax=ARM item.

Average of 76.65% of edges present in model also present in boots; 6.45% of edges not present in model but present in boots.

(5) Thailand

	1.	2.	3.	4.	5.	6.	7.	8.	9.	10.	11.	12.	13.	14.	15.	16.	17.	18.	19.	20.	21.	22.	23.	24.	25.	26.	27.
**R1**	-	82.5	31.1	4.6	60.9	58.9	9.5	0.1	29.1	40.3	0.1	0.1	12.1	0.4	0	9.2	0.9	4	1.2	0	0	0.1	0.3	7.2	0.1	81.5	0.4
**R2**		-	4.3	100	12.4	11.2	32.1	92	49.6	76.9	1.8	0.5	22.3	0.3	0.9	0.9	67.1	0	0	0.4	1.4	1.4	0	0	0.2	0	0
**R3**			-	62.1	51.1	8.3	4.5	81.7	0.4	86.1	0.2	0.4	0.1	0	1.9	0.5	0.7	0	0	11.3	0.7	1.6	29.2	37.3	0.5	49	0
**R4**				-	62.1	1.2	0.5	12.3	63.5	0.4	2	20.1	0	13.8	0	0	2.9	0	0.1	0	20.1	0.3	0.3	0.9	10.3	46.6	5.9
**R5**					-	12.8	39.2	0.1	3.1	0	2.1	9.5	98.3	0	2.6	0.2	9	1.6	0	0.1	2.6	0	38.2	0	9.4	0	46.4
**R6**						-	15.9	99.7	40.5	38.8	0.1	6	0	0.1	0.3	0.8	0.1	4.6	0.1	0	0.7	0	0.2	27	23.6	8.4	0
**R7**							-	5.5	48	0	0.4	0	1.4	0.2	0	7.8	0.2	7.2	4	21.2	0.1	0	0.1	3.9	0.8	0.3	0.9
**R8**								-	3.9	20.9	0	55.1	0	0.2	73	99.6	38.7	0	17.5	61.5	0.1	8.7	0	0.2	0.9	14.1	1.8
**R9**									-	82.3	3	7.6	7.5	0.1	0.4	0.8	0	0	0	0.2	3.6	0	12.4	21.8	3.1	0	0.3
**R10**										-	47	2.5	6.3	0	0	0.3	81.3	65.5	6.5	0.3	0.1	0.2	0.4	0	1.2	0.1	10.6
**A1**											-	7.8	39.1	9.9	17.7	95	2.3	35.1	33.6	99.8	0.6	44.9	0	9.4	0.1	13	0
**A2**												-	91.7	4.8	0	2	0.4	0	4	1.1	0.6	56.3	16.8	95.9	69.1	0.3	53.4
**A3**													-	6.5	5.7	49.7	0.2	9.5	56.5	0.1	21.9	0	0.4	27.1	0.6	87.5	0.1
**A4**														-	100	0	1.4	57.5	0.6	26.7	100	0.4	0	2.9	61.5	0.6	14.5
**A5**															-	54.8	4	21.8	0.8	0.9	74.1	6.4	0	66.1	80.5	9.9	29
**A6**																-	3.1	0	0.1	0	22.9	20.6	4.9	40.5	92	1.1	18.2
**A7**																	-	1	46	94	3.7	36.7	70.6	100	3.8	68.7	51.7
**A8**																		-	57.8	6.5	88.7	1.3	0	4.6	37	0.2	77.9
**A9**																			-	42.4	0	100	19.5	0.1	2.2	18.6	0.1
**A10**																				-	0	40.8	100	1	5.3	81	8
**A11**																					-	21.1	2.1	55.9	92.1	0	28.6
**A12**																						-	60.4	0	0.1	0	1.2
**A13**																							-	0.9	3.4	2.6	53.8
**A14**																								-	0.2	12.3	0
**A15**																									-	54.1	32.3
**A16**																										-	22.1
**A17**																											-

Note: Rx=RRM item; Ax=ARM item.

Average of 67.55% of edges present in model also present in boots; 10.50% of edges not present in model but present in boots.

(6) US

	1.	2.	3.	4.	5.	6.	7.	8.	9.	10.	11.	12.	13.	14.	15.	16.	17.	18.	19.	20.	21.	22.	23.	24.	25.	26.	27.
**R1**	-	6.7	41.5	0.1	0.6	99.3	94.4	8	17.6	78.1	0	0.1	0	48.1	25.2	1.9	64.9	6.8	0.2	29.9	0	0	0.3	0	0.5	72.1	9.5
**R2**		-	98.4	90.1	100	3.5	47.6	0.4	89.6	0	68.5	3.4	0.2	0	0	2.5	10.3	0	0	0	0.4	7.2	4.5	0.1	7.6	17.2	0.7
**R3**			-	30.8	37.9	0.3	67.8	11.9	54.8	71.1	0.9	14.9	81.8	0	0.3	2.2	20.7	0	0.1	0	0.2	1.5	0	1.7	0.1	11.2	3.1
**R4**				-	14.9	77.4	10	0.7	96.9	59.5	0.1	20.9	0.1	0	0.1	4	0	4.3	0.2	0.1	66	0	6.9	0	2	0	8
**R5**					-	48.2	17.3	82.4	33.5	42.5	3.3	0.5	3.9	0	2.4	0	14.8	0	0.2	0.1	0.7	0.8	0.1	0.7	0	0.1	0.1
**R6**						-	12.9	42	52.5	99.2	0.1	0	3.9	16	0	10	0.6	2.1	28	0	0	0.8	23.5	39.1	3.2	1.7	18.6
**R7**							-	0	0	0.1	0.6	0	1	0.1	0	0.1	0.1	6.7	0.1	63.1	1.6	19.5	59.2	0.4	63.1	0.2	0
**R8**								-	0	27.4	29.7	24.9	1.6	0	0	78.2	15.4	0.1	1.1	0.2	0.1	0	0	6	23.6	6.2	1.4
**R9**									-	7.9	25.5	49.3	1.8	0.9	0	0	0	0	6	14.8	0.3	2.2	17.4	1.3	0.4	0.1	0
**R10**										-	0	0	0	0	1.6	32.7	0.1	55	0.3	1.2	0	30.9	3.5	34.3	14.3	12.2	0.1
**A1**											-	74.9	97.5	55.6	10	0.5	1.5	7.9	0.4	1.1	28.3	35.2	6.4	84.4	24.7	36.5	0
**A2**												-	1.4	26.1	18.7	65.4	6.2	0	0.1	0	0.7	34.7	5.4	52.7	0.2	99.3	0
**A3**													-	17.9	0	0	62.7	20.9	0	0	0.2	0.3	47.7	63.5	8.3	59	0
**A4**														-	100	8.6	34.5	2.1	1.3	0.1	97.5	0.1	0.1	0	8.6	0.2	55.7
**A5**															-	8	49.1	25.4	6.4	9.6	20.1	0.2	0.1	1.3	51.9	0.1	14.7
**A6**																-	1.9	2.7	2.8	0.5	5.8	0	6.4	28.7	91.6	1.5	98.3
**A7**																	-	4.8	0.3	100	0.6	96.1	50.4	18.3	0.1	0.9	0.1
**A8**																		-	21.9	38.9	45	0	0.1	0.5	84	0.1	83.4
**A9**																			-	33.5	0.1	100	32.9	16.3	1.6	28.5	2.8
**A10**																				-	0	3.5	96.7	0.5	0.3	62.4	18.3
**A11**																					-	11.2	1.9	11	100	0.4	38.5
**A12**																						-	19.6	0	0.6	0.2	2.9
**A13**																							-	6	5.3	10.8	0
**A14**																								-	1.3	6.2	1.6
**A15**																									-	4.6	39.1
**A16**																										-	42.7
**A17**																											-

Note: Rx=RRM item; Ax=ARM item.

Average of 66.91% of edges present in model also present in boots; 7.02% of edges not present in model but present in boots.

(7) Vietnam

	1.	2.	3.	4.	5.	6.	7.	8.	9.	10.	11.	12.	13.	14.	15.	16.	17.	18.	19.	20.	21.	22.	23.	24.	25.	26.	27.
**R1**	-	4.9	6.8	82.7	3.9	97.3	2.3	99.5	27	99.4	0	18	31.6	0.4	0	23.8	0	0	0	2.7	8.1	0.1	0	0	0	0	0
**R2**		-	81.2	76.8	18.2	0	0.7	0	99	37.5	0.9	1.9	5.6	13.6	1.7	7.8	14	0.3	0.1	0.8	4.9	2.3	0.3	68	0.2	5.3	0.1
**R3**			-	4.5	98.6	0	46.3	99.6	99	5.3	2.2	0	0.1	0.1	0.1	7.5	0.1	3.8	0.2	28.6	0	0.1	1.7	0	0	62.3	0.1
**R4**				-	12.5	93	1.8	0.1	99.8	0.3	75.2	0	1.7	2	0.1	9.5	0	0.4	1.5	2.1	0.2	0.1	0.1	0.1	0.1	0.7	0.1
**R5**					-	46.8	1.2	45.8	83.6	71.4	0.5	23.5	92.2	1.6	0.7	0.1	0	0	2.4	0.7	0	0.5	0	1	0	0.2	0
**R6**						-	30.2	39.1	34.9	83.7	2.3	1.1	4.4	0	0.4	45.4	12.1	0.4	0.1	0	61.5	0	5	0	0	0	0
**R7**							-	0.2	18.6	0	0.1	9.2	45.2	0	59.4	7.6	1.2	0	13.6	4.9	0.1	2.7	0	0.3	0	0.4	0
**R8**								-	0.4	3	0.5	1.6	0.1	0.1	0	4.9	0	60	0.7	0	2	0	0.6	25	0.1	30.5	30.4
**R9**									-	69.7	0	0.1	42.6	0.9	14.4	0	0	0	2.5	0.3	8.4	0	0.1	3.4	0	1.2	0
**R10**										-	6.2	0.3	0.2	74.5	1.4	0	0.1	0.3	9.8	0.9	2.9	0	82.2	11.2	0.1	14.4	0.3
**A1**											-	46	88.4	0.4	0.6	22.6	50.9	11.4	75.2	72.2	5.1	6.6	0	0.5	0	14	0
**A2**												-	0.1	13.4	0	7.3	0.7	0	0.3	0.1	0	0	7.6	22.8	0	3.8	73.9
**A3**													-	8.6	3	0.4	84.4	21.3	0.2	0	0	0.7	46.8	95.9	15.9	97.6	1.5
**A4**														-	100	25.6	0	0.2	0	0.2	100	0	5.7	7.2	98	18.5	89
**A5**															-	0.7	0	99.8	5.4	1	98.1	92.3	2	0.2	23	0.1	95.3
**A6**																-	0.1	0.2	1.1	17.2	87	0	0.5	59.3	23	24.2	0.3
**A7**																	-	96.3	27.1	91.1	0	55.1	74.6	2.5	2.4	21.4	0
**A8**																		-	13.2	38.9	14.2	0	0	0.1	38.1	75.3	96.8
**A9**																			-	89.6	24.9	100	0	2.7	0.7	6.6	0.1
**A10**																				-	50.3	7.9	96.8	10.7	1.8	0.1	45.9
**A11**																					-	0.1	1.1	6.4	100	0.3	0.8
**A12**																						-	100	1.9	54.5	0.1	5.9
**A13**																							-	9.6	0	0.5	34.5
**A14**																								-	0.1	27.2	0.1
**A15**																									-	0.4	96.2
**A16**																										-	0.3
**A17**																											-

Note: Rx=RRM item; Ax=ARM item.

Average of 75.37% of edges present in model also present in boots; 5.56% of edges not present in model but present in boots.

Table S4. Results of the Bayesian posterior predictive check test (structural difference test) for each pair of countries.

	China	Indonesia	Russia	Thailand	US	Vietnam
Brazil	1.633	1.915	1.968	1.995	1.685	1.608
China		1.891	1.753	1.716	1.645	1.463
Indonesia			2.223	1.847	1.785	1.650
Russia				2.120	1.887	2.005
Thailand					1.605	1.511
US						1.603

Note: Numbers indicate magnitude of Jensen-Shannon divergence (Kullback-Leibler divergence). All comparisons were significant at $p<.001$.

Table S5. Measurement invariance and alignment tests

	df	χ^2	CFI	TLI	RMSEA	RMSEA CI_{90}	SRMR	ΔCFI
RRM								
Configural invariance	245	434.10	.997	.997	.035	.029, .040	.032	-
Metric invariance	299	372.17	.997	.997	.020	.012, .026	.039	<.01
Scalar invariance	353	661.23	.988	.990	.037	.033, .041	.050	<.01
Alignment: R^2 loadings = .996; R^2 intercepts = .999; % of item parameters non-invariant: Nu = 4.3; Lambda = 0.								
ARM								
Configural invariance	833	3964.43	.982	.979	.077	.074, .079	.058	-
Metric invariance	929	2304.61	.980	.979	.048	.046, .051	.060	<.01
Scalar invariance	1025	3171.77	.968	.971	.057	.055, .060	.069	-.012
Alignment: R^2 loadings = .993; R^2 intercepts = .999; % of item parameters non-invariant: Nu = 10.1; Lambda = 0.								

Note: CFI=comparative fit index, TLI=Tucker-Lewis index, RMSEA=Root mean square error of approximation, SRMR=standardized root mean square residual.

Invariance and multi-group factor analysis alignment tests were conducted following the recommendations of Fischer and Karl (2019). In their guide they argue that because scalar invariance is rarely achieved, additional alignment tests can offer greater insight. They follow the criteria recommend by Asparouhov and Muthén (2014) who suggest that R2 values close to 1 to indicate general invariance, and that the percentage of non-invariant loading and intercept parameters be below 25%.

Fischer, R., & Karl, J. A. (2019). A primer to (cross-cultural) multi-group invariance testing possibilities in R. *Frontiers in Psychology, 10*. https://doi.org/10.3389/fpsyg.2019.01507.
